# Involvement of a NIMA-related kinase in cell division of the liverwort *Marchantia polymorpha*

**DOI:** 10.1093/pcp/pcaf021

**Published:** 2025-02-17

**Authors:** Hikari Mase, Aoi Sumiura, Yoshihiro Yoshitake, Takayuki Kohchi, Taku Takahashi, Hiroyasu Motose

**Affiliations:** Department of Biological Science, Graduate School of Natural Science & Technology, Okayama University, Tsushimanaka 3-1-1, Okayama 700-8530, Japan; Department of Biological Science, Graduate School of Natural Science & Technology, Okayama University, Tsushimanaka 3-1-1, Okayama 700-8530, Japan; Graduate School of Biostudies, Kyoto University, Kitashirakawa-oiwakecho, Sakyo, Kyoto 606-8502, Japan; Graduate School of Biostudies, Kyoto University, Kitashirakawa-oiwakecho, Sakyo, Kyoto 606-8502, Japan; Department of Biological Science, Graduate School of Natural Science & Technology, Okayama University, Tsushimanaka 3-1-1, Okayama 700-8530, Japan; Department of Biological Science, Graduate School of Natural Science & Technology, Okayama University, Tsushimanaka 3-1-1, Okayama 700-8530, Japan

**Keywords:** estradiol, *Marchantia polymorpha*, NIMA-related kinase, rhizoid, thallus, tip growth

## Abstract

**Never-in-mitosis A (NIMA)-related kinases (NEKs) regulate a series of mitotic events in fungi and animals, whereas plant NEKs have been shown to control the growth direction of cells and organs. Plant NEKs are highly expressed in the meristem, but whether they regulate meristematic activity remains unknown. The liverwort *Marchantia polymorpha* has a single functional Mp*NEK1* gene, and its knockout results in twisted rhizoid growth. For a gain-of-function approach, we generated lines for the inducible expression of Mp*NEK1* using an estrogen receptor-mediated system. Estradiol treatment effectively induced the accumulation of Mp*NEK1* mRNA and MpNEK1–Citrine fusion protein throughout the plant. MpNEK1 overexpression severely suppressed rhizoid and thallus growth, ultimately leading to the lethality of juvenile plants. This severe effect was observed even at the nanomolar level of estradiol. EdU (5-ethynyl-2ʹ-deoxyuridine) staining and microtubule imaging clearly indicated suppression of cell division by estradiol-induced MpNEK1. MpNEK1 induction also reduced cortical microtubule density and dynamics but did not severely affect cell growth and morphology in thalli. Overexpression of kinase-deficient MpNEK1 also suppressed thallus and rhizoid growth, although to a slightly lesser extent than wild-type MpNEK1, indicating a phosphorylation-independent mechanism of growth suppression. Furthermore, Mp*nek1* mutants exhibited growth suppression in their reproductive organs, the gametangiophores. This supports the role of MpNEK1 in cell division, as observed in both fungi and animals**.

## Introduction

Never-in-mitosis A (NIMA)-related kinase (NEK) is a Ser/Thr protein kinase that controls a series of mitotic events in fungi and animals (O’Connell et al. 2003, Fry et al. 2012), while plant NEK members have been shown to regulate directional growth (Motose et al. 2008, Sakai et al. 2008). *Arabidopsis* NEK6 suppresses ectopic outgrowth of epidermal cells through microtubule organization and interaction with other NEK members (Motose et al. 2011). NEK6 localizes to the shrinking ends of microtubules, phosphorylates five amino acid residues of β-tubulin, and depolymerizes aberrant cortical microtubules to promote anisotropic growth of epidermal cells (Takatani et al. 2017). Furthermore, NEK6 has been shown to stabilize the growth direction of hypocotyls by selectively removing cortical microtubules that best align with maximal tensile stress (Takatani et al. 2020). NEK6-mediated attenuation of tensile stress response increases the noise in microtubule orientation to buffer mechanical conflicts and stabilize growth direction.

The liverwort *Marchantia polymorpha* is an emerging model to study the evolution of land plants (Kohchi et al. 2021) because of its low genetic redundancy (Bowman et al. 2017) and various genetic tools (Ishizaki et al. 2015, 2016). Indeed, *Arabidopsis* has seven NEK members, whereas *M. polymorpha* has a single *NEK* gene, Mp*NEK1* (Takatani et al. 2015, Otani et al. 2018). Thanks to these advantages, MpNEK1 has been shown to direct tip growth of rhizoids through microtubule depolymerization in the apical region (Otani et al. 2018). Rhizoids are filamentous rooting cells, mainly elongating from the ventral epidermis and are thought to be required for plant anchorage to the soil and water/nutrient uptake (Jones and Dolan 2012, Shimamura 2016, Kohchi et al. 2021, Kanda et al. 2022). Mp*nek1* mutant rhizoids frequently change their growth direction, resulting in the zigzag morphology (Otani et al. 2018) and the defect of invasive growth to enter the hard substratum (Mase et al. 2022). MpNEK1 localization in the apical microtubule foci and the increased microtubule stability in Mp*nek1* mutants demonstrate that MpNEK1 reorganizes and destabilizes apical microtubules to maintain the growth polarity of rhizoids (Otani et al. 2018). A similar phenotype was observed in the mutants of a microtubule-associated protein, WAVE DANPENED-LIKE (MpWDL) (Honkanen et al. 2016, Champion et al. 2021). MpWDL might stabilize longitudinal microtubules in the shank region of rhizoids. Thus, microtubule organization and dynamics are essential for the directional growth of rhizoids.

However, it is not clear how MpNEK1 regulates microtubules to stabilize growth direction. Furthermore, the functional significance of Mp*NEK1* expression in the meristem remains unclear. The gain-of-function approach could be helpful to elucidate these problems and identify downstream proteins that are phosphorylated by MpNEK1. In this study, we used an estradiol-inducible system to overexpress Mp*NEK1* in *M. polymorpha* and investigate its effects on plant growth and development.

## Results

### Establishment of Mp*NEK1* inducible lines

In this study, we generated Mp*NEK1*-inducible transgenic lines using the XVE chimeric transcriptional activator (Zuo et al. 2000). This activator is composed of the DNA-binding domain of LexA, the transcriptional activation domain of VP16, and the estradiol-binding domain of the human estrogen receptor. This system has no apparent toxic physiological effects and has been effectively used in flowering plants. The XVE vector was optimized for the application to *M. polymorpha* in several points. Because the endogenous elongation factor Mp*EF1α* promoter is more active in the meristem and reproductive organ than the cauliflower mosaic virus *35S* promoter (Althoff et al. 2014), the Mp*EF1α* promoter was utilized to drive XVE. XVE system has been applied in several studies in *M. polymorpha* (e.g. Flores-Sandoval et al. 2016, Furuya et al. 2022, Bao et al. 2024) but not fully characterized.

We isolated eight transgenic lines by transformation of wild-type (WT) sporelings with pMpGWB344-MpNEK1 (MpEF1α pro:XVE≫LexAop:MpNEK1), in which XVE is expressed under the control of Mp*EF1α* promoter and activates transcription of Mp*NEK1* through the binding to the LexA operator in the presence of estradiol. Among them, seven transgenic lines (Lines #2–8) were almost identical to the WT in the growth and morphology of thalli. We examined Mp*NEK1* expression in these transgenic lines by quantitative real-time polymerase chain reaction (RT-qPCR) (Supplementary Fig. S1). Two-week-old thalli of the WT and transgenic lines were transferred to the medium with or without β-estradiol and incubated for 24 hours. Mp*NEK1* expression remarkably increased in most transgenic lines (#2, 4, 5, 7, and 8) by a factor of 4–15 in the estradiol treatment than in the estradiol-free medium. Estradiol did not induce Mp*NEK1* expression in the WT and Line #6. Mp*NEK1* induction was reproducible and reliable, especially in Lines #2, 4, and 8, which exhibited no obvious defects in growth and morphology without estradiol. Thus, these lines were used for further analyses.

### Mp*NEK1* induction severely suppresses thallus growth

**Figure 1.**
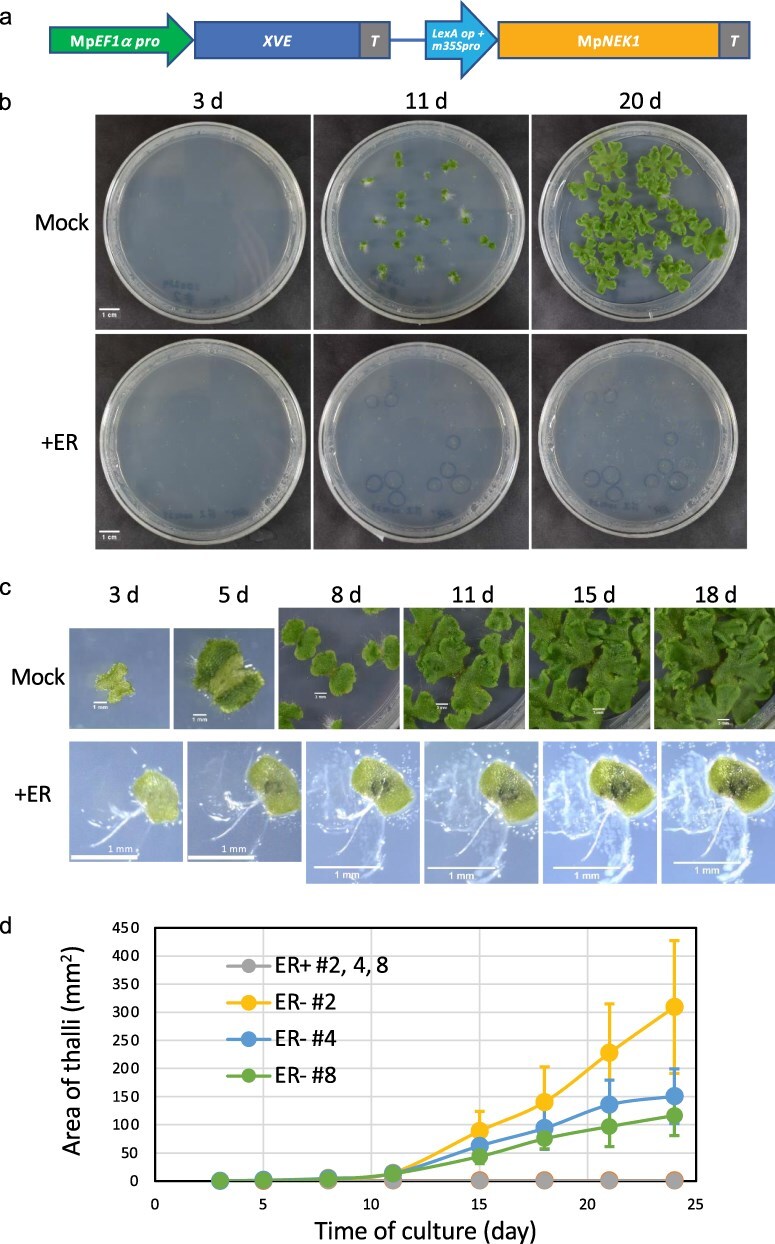
Effect of estradiol on thallus growth of Mp*NEK1* inducible line. (a) Construct for Mp*NEK1* induction. Mp*EF1a* promoter, terminator (T), LexA operator (LexA op), minimal CaMV35S promoter (m35Spro). The diagram is not shown to scale. (b) The gemmae of Mp*NEK1*-inducible line (#2) were planted in the agar medium with (+ER) or without 10 µM estradiol (Mock) and grown for 3, 11, and 20 days. (c) Time course of growth of Mp*NEK1*-inducible line (#2) with (+ER) or without 10 µM estradiol (Mock). (D) Quantification of thallus growth of Mp*NEK1*-inducible line (#2, 4, 8) with (ER+) or without 10 µM estradiol (ER−). The mean projection area of thalli (*n* = 8–11 plants) was quantified by ImageJ. Circles and error bars indicate mean values and standard deviations, respectively.

We conducted a phenotypic analysis to assess the effects of estradiol-induced MpNEK1 on growth and development. The gemmae of the WT and Mp*NEK1*-inducible lines were grown in the presence or absence of exogenous β-estradiol for 24 days. There was no obvious effect of β-estradiol on the growth and morphology of the WT thalli (Supplementary Fig. S2). In the inducible lines, thallus growth was severely suppressed when cultured with β-estradiol (Fig. 1). Most gemmalings stopped growing and died in the presence of estradiol (Fig. 1), whereas the non-inducible line #6 did not show growth retardation (Supplementary Fig. S3). These results clearly demonstrate that the induction of Mp*NEK1* severely suppresses thallus growth and causes juvenile lethality.

We next examined whether the effect of Mp*NEK1* induction is reversible or not. The gemmalings of the WT and Mp*NEK1*-inducible lines were grown in the presence of estradiol and then transferred to the estradiol-free medium at various times after estradiol treatment (Supplementary Fig. S4). After 1- and 3-day treatment with estradiol, thallus growth seemed to be recovered, whereas irreversible growth suppression was observed after 7- and 14-day treatment. In the WT, the growth inhibitory effect of estradiol was not observed. Therefore, 1-week Mp*NEK1* induction (estradiol treatment) confers an irreversible effect on thallus growth.

**Figure 2.**
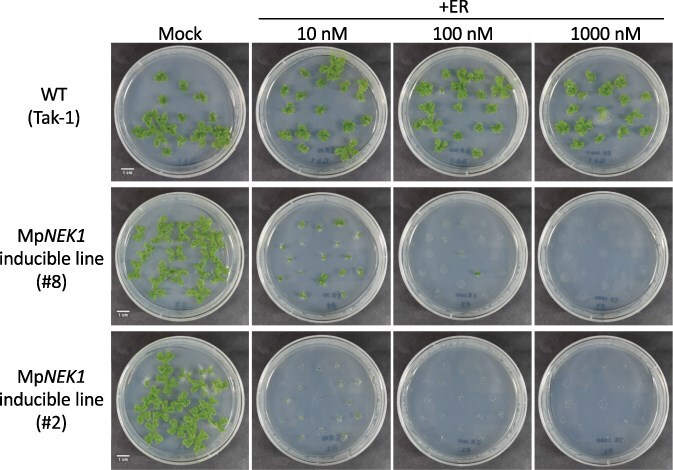
Effect of various concentrations of estradiol on thallus growth. The gemmae of the WT and Mp*NEK1*-inducible lines were planted in the agar medium supplemented with or without estradiol at concentrations of 10, 100, or 1000 nM and grown for 16 days.

In the XVE induction system in flowering plants, 2–10 µM concentrations of estradiol have been commonly used. In the Mp*NEK1*-inducible lines, 10 µM estradiol caused severe growth suppression and lethality. However, more mild conditions would be desirable to further investigate the MpNEK1 function. To determine the less-effective conditions and dose-dependent effect of estradiol, thalli of the WT and Mp*NEK1*-inducible lines were incubated in the presence of estradiol at various concentrations (Fig. 2, Supplementary Fig. S5). The WT thallus showed no obvious phenotype in all concentrations tested, whereas thallus growth was suppressed in the Mp*NEK1*-inducible lines even at 10 and 100 nM of estradiol. From this result, Mp*NEK1* induction has a severe effect on thallus growth.

**Figure 3.**
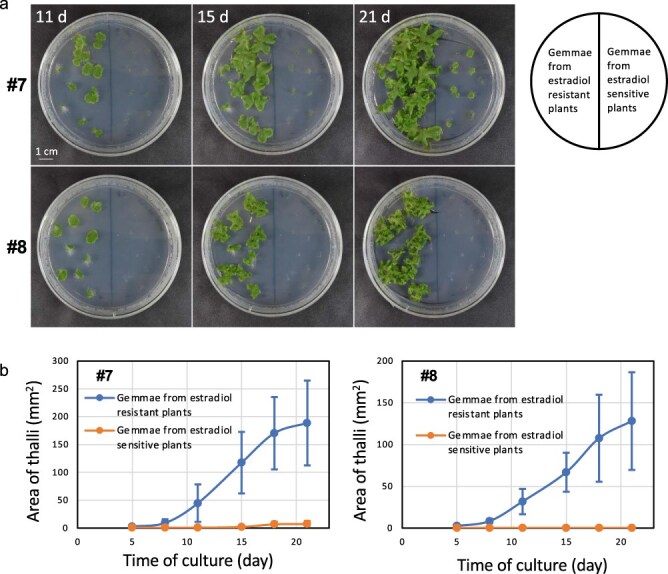
Estradiol resistance and its transfer to the next generation. (a) The gemmae of the estradiol-resistant plants or sensitive plants of Mp*NEK1*-inducible lines were planted in the agar medium supplemented with 10 µM estradiol and grown for 11, 15, and 21 days. (b) Quantification of growth of gemmalings derived from the estradiol-resistant plants or -sensitive plants of Mp*NEK1*-inducible lines. The mean projection area of thalli (*n* = 10 plants) was quantified by ImageJ. Circles and error bars indicate mean values and standard deviations, respectively.

We noticed that some plants obtained the resistance to estradiol and survived in the agar medium supplemented with estradiol (Fig. 3). The estrogen resistance was observed in the next-generation gemmae, suggesting the inheritance of gene silencing from the thallus to gemma. These estradiol-resistant plants appeared more frequently at lower concentrations and exhibited no induction of Mp*NEK1* (Supplementary Fig. S6). At the lower concentrations, larger numbers of thallus cells could survive and acquire estradiol resistance. Thus, lower concentrations of estradiol create an undesirable condition that can lead to estradiol resistance.

### MpNEK1 induction suppresses cell proliferation

**Figure 4.**
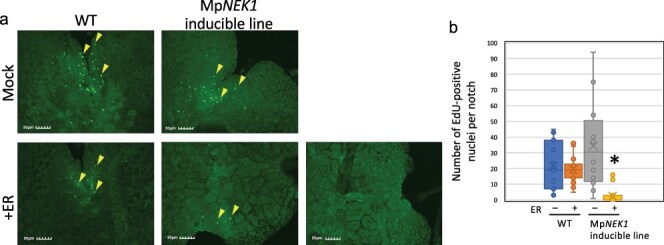
Effect of estradiol on cell proliferation. (a) EdU-labeled nuclei in the gemmalings treated with 1 µM estradiol for 4 days. The gemmae of the WT and Mp*NEK1*-inducible line were planted in the medium supplemented with or without 1 µM estradiol, grown for 4 days, and incubated with 10 µM EdU for 1 hour in the same kind of liquid medium. EdU-labeled nuclei were visualized according to the manufacturer’s instructions as described in methods. (b) Quantification of EdU-labeled nuclei in the thalli grown as in (a). Data are shown by box plots (*n* = 12–16 plants). An asterisk indicates significant difference from the control (−ER) (*t*-test, *P* < .05).

Previous studies show that plant NEKs mainly regulate the growth direction of cells and organs. However, severe suppression of thallus growth by estradiol-induced Mp*NEK1* suggests some roles of plant NEK in cell division and proliferation. To determine whether estradiol-induced Mp*NEK1* affects cell division, we monitored cell proliferation activity by staining with EdU (5-ethynyl-2ʹ-deoxyuridine) (Fig. 4, Supplementary Fig. S7). This thymidine analog can be incorporated into DNA during the S phase. The subsequent reaction of EdU with a fluorescent azide labels newly replicated DNA strands of the S-phase cells during the incubation with EdU.

In both the WT and inducible lines, EdU-positive nuclei were observed in the meristematic notch region in thalli grown in the absence of estradiol (Fig. 4, Supplementary Fig. S7). The number of EdU-positive nuclei was not affected in the WT by estradiol, whereas it was significantly reduced in the estradiol-treated inducible lines. Hence, estradiol-induced Mp*NEK1* severely suppressed cell proliferation.

### MpNEK1 induction suppresses mitotic cell division

**Figure 5.**
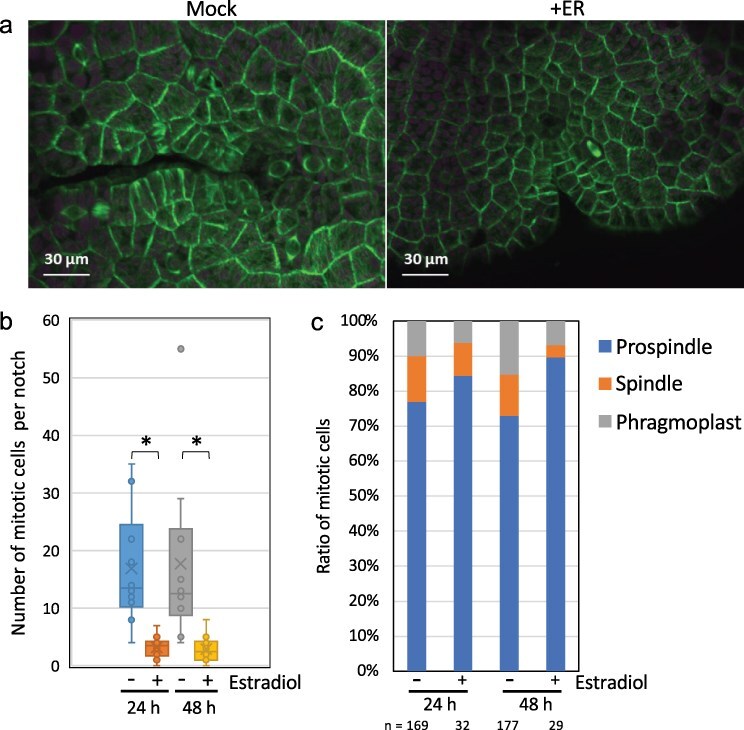
Effect of MpNEK1 induction on mitosis. (a) Mitotic cells in the apical notches. The gemmae of MpNEK1-inducible line with a microtubule marker CaMV35S:Citrine–MpTUB2 were grown in the estradiol-free medium for 3 days and then supplemented with the liquid medium with (+ER) or without 10 µM estradiol (Mock). Thalli were observed under a confocal microscope after 24 hours of treatment. Green: Microtubule, magenta: plastid autofluorescence. (b) Number of mitotic cells per notch in the MpNEK1 inducible line with CaMV35S:Citrine–MpTUB2 after 24 and 48 hours of treatment with or without estradiol. Data are shown in the box plot (*n* = 10 notches, *N* > 6 plants). Asterisks indicate significant difference (*t*-test, *P* < .01). (c) The classification of mitotic cells with prospindle, spindle, or phragmoplast (*n* indicates the number of mitotic cells used for the classification).

Because *Arabidopsis* NEK6 depolymerizes specific microtubules, which detached from the plasma membrane or aligned to the maximal tensile stress, to regulate cell expansion and organ growth (Takatani et al. 2017, 2020), we monitored the effect of MpNEK1 overexpression on microtubule organization and mitotic apparatus. MpNEK1-inducible construct was introduced into the transgenic lines expressing a microtubule marker Citrine–MpTUB2 to generate the inducible lines expressing Citrine–MpTUB2. The gemmae of these transgenic plants were grown for 3 days in the estradiol-free medium and then applied to the liquid medium with or without 10 µM estradiol. After 24 and 48 hours of treatment, thallus cells were observed with confocal microscopy. In the estradiol-treated thalli, the number of mitotic cells was significantly decreased compared with the mock-treated thalli (Fig. 5a and b, Supplementary Fig. S8). We classified mitotic cells according to mitotic structures, prospindle, spindle, and phragmoplast since these structures are clearly distinguished under confocal microscopy (Buschmann et al. 2016). We observed a slight increase of cells with prospindle and a slight decrease of cells with spindle and phragmoplast (Fig. 5c), suggesting that MpNEK1 induction has a mild effect on mitotic progression (e.g. transition from prospindle to spindle formation). In summary, overexpression of MpNEK1 significantly suppressed mitotic cell division.

### MpNEK1 induction reduces microtubule density and dynamics

**Figure 6.**
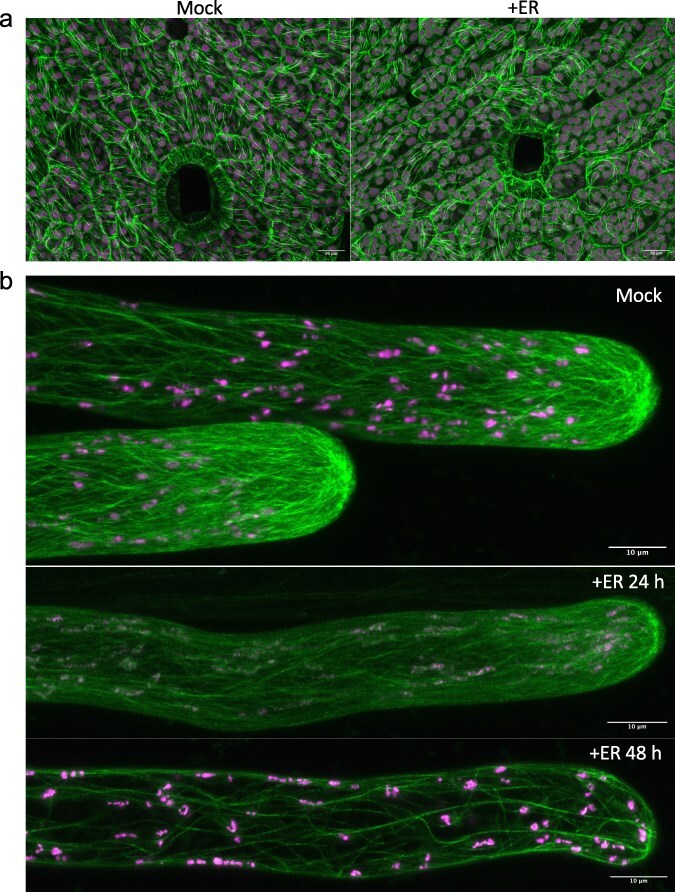
Effect of MpNEK1 induction on microtubules. (a) Cortical microtubules in thallus epidermal cells. The gemmae of MpNEK1 inducible line with CaMV35S:Citrine–MpTUB2 were grown in the estradiol-free medium for 6 days and then supplemented with the liquid medium with (+ER) or without 10 µM estradiol (Mock). Thalli were observed under a confocal microscope after 24 hours of treatment. Green: Microtubule, magenta: plastid autofluorescence. (b) Microtubules in rhizoids. The gemmae of MpNEK1-inducible line with CaMV35S:Citrine–MpTUB2 were grown as in (a). Rhizoids were observed under a confocal microscope after 24 and 48 hours of treatment. Green: microtubule, magenta: plastid autofluorescence.

We next analyzed the effects of MpNEK1 induction on cell growth and cortical microtubule organization (Fig. 6). Estradiol treatment did not obviously affect the morphology of thallus epidermal cells but seemed to disorganize cortical microtubules (Fig. 6a). These were confirmed by the quantification of parameters of epidermal cells and microtubules (**Table 1**). Cell area and morphological parameters were not severely affected by estradiol treatment but both bundling and density of cortical microtubules were decreased. The quantification of the dynamic parameters of cortical microtubules showed that MpNEK1 induction decreased growth rate, catastrophe events, and dynamicity of microtubule plus ends (**Table 2**). These results show that MpNEK1 induction decreases microtubule density and dynamics in the thallus.

**Table 1.** Parameters of cortical microtubules and cell size/morphology

	Mock (*n* = 103)	+Estradiol (*n* = 121)
Microtubule		
Bundling (skewness)	1.63 ± 0.29	1.52 ± 0.24*
%Occupancy	4.88 ± 0.72	3.79 ± 0.78**
Cell		
Area (µm^2^)	690.2 ± 183.6	694.9 ± 235.4
Circularity	0.72 ± 0.09	0.72 ± 0.10
Aspect ratio	2.00 ± 0.60	2.11 ± 0.71
Roundness	0.54 ± 0.14	0.52 ± 0.16

^*,**^ Asterisks indicate significant difference from the control (Student’s *t*-test.

* *P* < .005,

** *P* < .0001, *n* = number of cells).

**Table 2.** Parameters of plus-end dynamics of cortical microtubules

Parameters	Mock (*n* = 52)	+Estradiol (*n* = 50)
Growth (µm/min)	3.63 ± 1.45	2.35 ± 1.12***
Shrinkage (µm/min)	6.96 ± 2.48	6.01 ± 2.96
Catastrophe (events/sec)	0.022 ± 0.026	0.011 ± 0.007*
Rescue (events/sec)	0.032 ± 0.027	0.030 ± 0.052
Dynamicity (µm/min)	3.75 ± 2.19	2.55 ± 2.14**
Time spent (%)		
Growth	39.5	47.1
Pause	35.4	33.1
Shrinkage	25.1	19.4

^*,**,***^ Asterisks indicate significant difference from the control (Student’s *t*-test.

* *P* < .02,

** *P* < .01,

*** *P* < .001, *n* = number of cortical microtubules).

We also analyzed the effects of MpNEK1 induction on microtubule organization in rhizoids (Fig. 6b). In the control condition, most microtubules in the shank region aligned along the longitudinal direction and exhibited cable-like bundles, whereas microtubule foci were observed at the apical dome of rhizoids (*n* = 16/16, 16 rhizoids with microtubule foci/all 16 rhizoids). After 24 hours of estradiol treatment, microtubule signal was reduced but microtubule foci still existed in ∼70% of rhizoids (*n* = 12/17). After 48 hours of estradiol treatment, microtubules were further reduced and microtubule foci were absent in 80% of rhizoids (*n* = 3/15, 3 rhizoids with microtubule foci/all 15 rhizoids). The cable-like bundles still existed and extended toward the apical region. Concomitant with the lack of microtubule foci, rhizoids grow more wavy after 24–48 hours of estradiol treatment. Thus, MpNEK1 induction suppressed microtubule foci at the apical dome of rhizoids.

### The overaccumulation of MpNEK1–Citrine suppresses thallus growth

**Figure 7.**
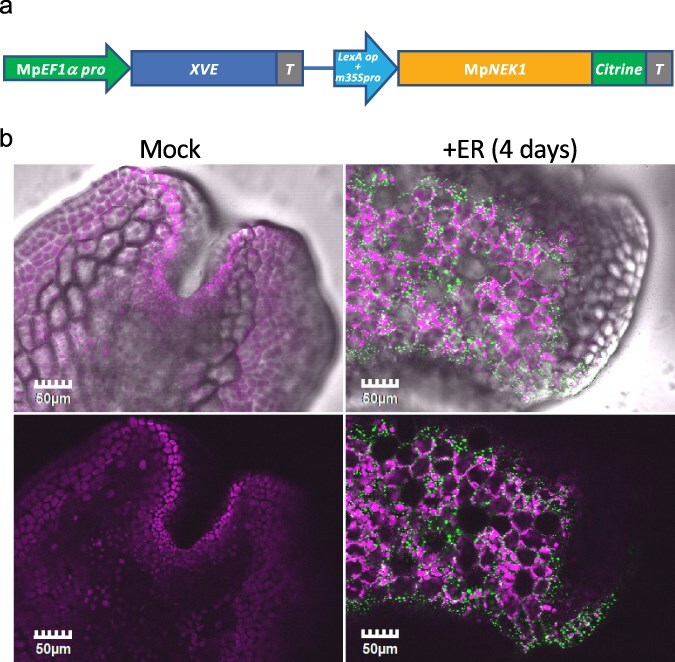
Localization of estradiol-induced MpNEK1–Citrine in the thallus. (a) Construct for Mp*NEK1* induction. Mp*EF1a* promoter, terminator (T), LexA operator (LexA op), and minimal CaMV35S promoter (m35Spro). The diagram is not shown to scale. (b) The gemmae of the Mp*NEK1*-inducible line were planted in the medium with (+ER) or without 1 µM estradiol (Mock), grown for 4 days, and observed under a confocal microscope. Upper panels are light field images merged with confocal images shown in the lower panels. Green: MpNEK1–Citrine, magenta: plastid autofluorescence.

We generated inducible lines of MpNEK1–Citrine fusion protein (Fig. 7), in which protein induction and localization could be easily detected by confocal microscopy, facilitating functional analysis of MpNEK1. Eight transgenic lines were isolated by the transformation of WT sporelings with pMpGWB144–MpNEK1–Citrine. Among them, four transgenic lines (Lines #1, 5, 7, and 8) were almost identical to the WT in the absence of estradiol. When grown in the agar medium supplemented with estradiol, these transgenic lines exhibited severe growth retardation and juvenile lethality (Supplementary Fig. S9).

We next analyzed the expression and localization of the MpNEK1–Citrine fusion protein (Fig. 7b, Supplementary Fig. S10A). The gemmae of MpNEK1–Citrine-inducible lines were grown for 4 days in the medium with or without 1 µM β-estradiol and observed under a confocal microscope (Fig. 7b). MpNEK1–Citrine was not detected in the absence of estradiol, whereas it was accumulated throughout estradiol-treated thalli (Fig. 7b, Video S1). MpNEK1–Citrine localized in the cytoplasmic particles. In the 24-hour estradiol treatment, similar expression and localization pattern were observed (Supplementary Fig. S10A). From these results, MpNEK1–Citrine was induced throughout the thallus within 24 hours after estradiol treatment and severely suppressed thallus growth.

We noticed an accumulation of MpNEK1–Citrine particles in the meristem in some transgenic plants even in the absence of estradiol (Supplementary Fig. S10B). This leaky expression level varied in each individual and transgenic line. In this study, we used plants and transgenic lines (Lines #1, 5, 7, and 8) that exhibited reduced leaky expression of MpNEK1–Citrine.

### The overaccumulation of MpNEK1–Citrine suppresses rhizoid growth

**Figure 8.**
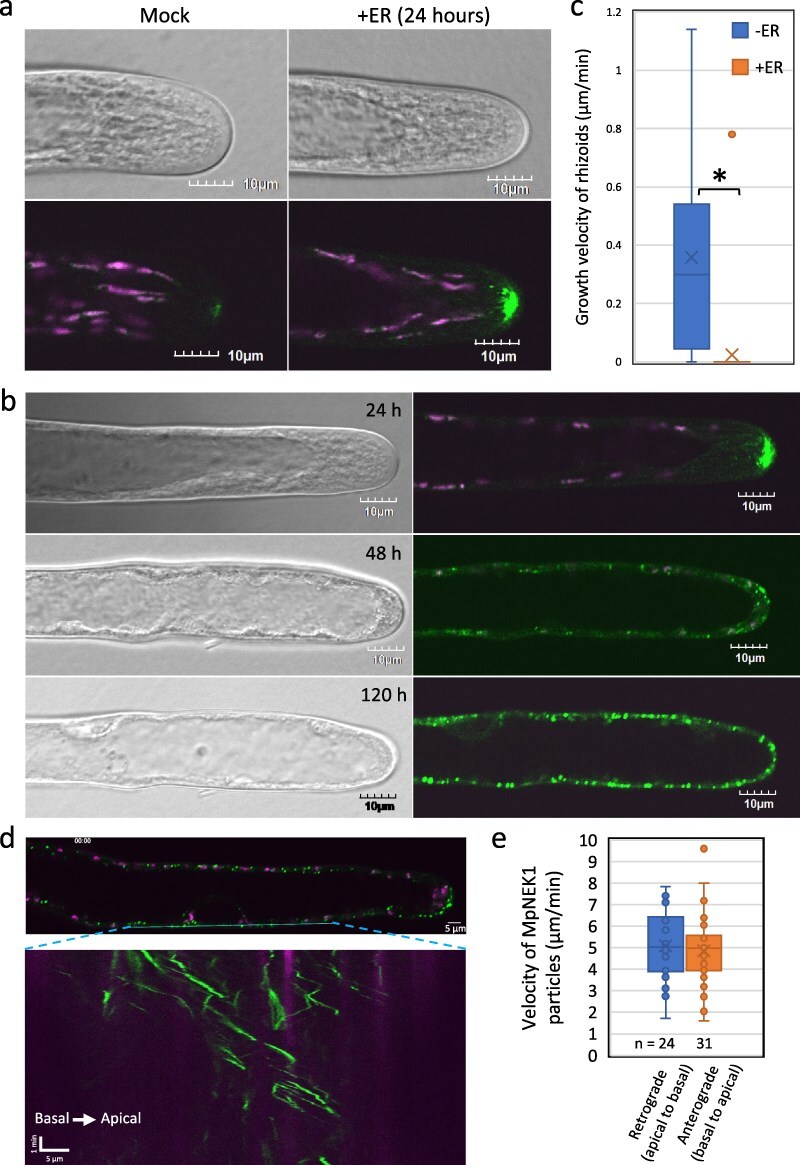
Localization of estradiol-induced MpNEK1–Citrine in rhizoids. (a) The gemmae of the Mp*NEK1*-inducible line were planted in the medium without estradiol, grown for 3 days, and then supplemented with (+ER) or without 1 µM estradiol (Mock). Rhizoids were observed under a confocal microscope. Green: MpNEK1–Citrine, magenta: plastid autofluorescence. (b) The gemmae of the Mp*NEK1*-inducible line were grown and treated with estradiol as in (a) and observed at the time indicated in each panel. The same rhizoid was observed under a confocal microscope over time. Green: MpNEK1–Citrine, magenta: plastid autofluorescence. (c) Growth velocity of rhizoids in the MpNEK1–Citrine-inducible line incubated for 48 hours with (+ER) or without 1 µM estradiol (−ER). Data are shown by box plots (*n* = 32 rhizoids). An asterisk indicates significant difference (*t*-test, *P* < .01). (d) Movement of MpNEK1–Citrine particles in rhizoids. The gemmae of the Mp*NEK1*-inducible line were grown and treated as in (b). Rhizoids were observed under a confocal microscope after 48 hours. The lower panel is a kymograph of MpNEK1–Citrine particles. Green: MpNEK1–Citrine, magenta: plastid autofluorescence. (e) Velocity of MpNEK1–Citrine particles. Data are shown by box plots (*n* = 24 or 31 particles). There was no significant difference (*t*-test, *P* > .05).

Taking account that MpNEK1 localizes in the apical microtubule foci to direct tip growth of rhizoids (Otani et al. 2018), we analyzed the localization of estradiol-induced MpNEK1–Citrine in tip-growing rhizoids (Fig. 8). Because rhizoids were not formed when gemmae were grown with estradiol at the start of culture, we added estradiol to 3-day-old gemmalings that had been grown without estradiol to investigate the localization and effects of MpNEK1–Citrine over time. After 24-hour treatment, MpNEK1–Citrine accumulated in the apical region of the rhizoids (Fig. 8a, Video S2). MpNEK1–Citrine expression varied in each rhizoid. A few rhizoids showed highly accumulated MpNEK1–Citrine, which formed large droplets, moved to the apical direction, and fused with each other (Video S3). To determine when MpNEK1–Citrine starts to accumulate, we observed rhizoids at the early periods of estradiol treatment (Supplementary Fig. S10C). MpNEK1–Citrine began to accumulate at ∼1 hour of treatment and gradually increased in the rhizoid apex. After 48 and 120 hours, MpNEK1–Citrine accumulated throughout the entire region of rhizoids in the particle and filamentous patterns (Fig. 8b, Videos S4–S7). Tip growth ceased or severely reduced after 48 and 120 hours of estradiol treatment (Fig. 8c, Videos S4–S6). After 48 hours, movement of MpNEK1–Citrine particles was obvious (Fig. 8d, Videos S4 and S5). MpNEK1–Citrine particles moved toward the apex (anterograde) and basal region (retrograde) at the same velocity (Fig. 8e). These results suggest that overexpression of MpNEK1 causes its mislocalization throughout the entire length of rhizoids to suppress rhizoid growth.

### The overexpression of kinase-deficient Mp*NEK1* suppresses growth of thalli and rhizoids

**Figure 9.**
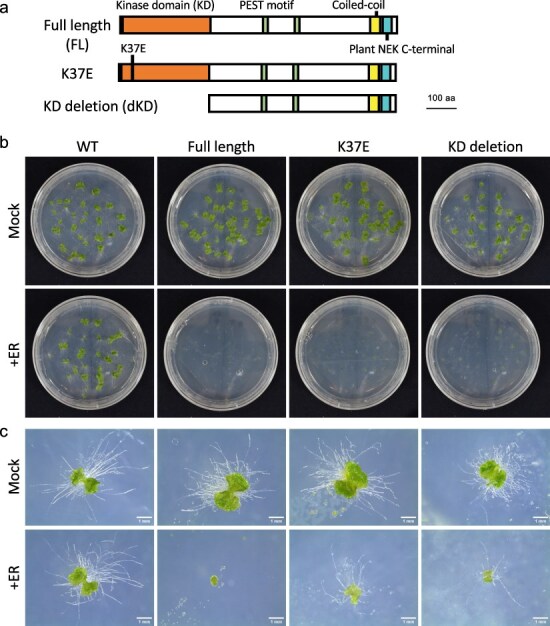
Effect of kinase-deficient MpNEK1 induction on the growth of thalli and rhizoids. (a) Full-length and kinase-deficient MpNEK1 used for estradiol induction. (b) The gemmae of the WT and inducible lines of full-length MpNEK1, MpNEK1^K37E^, or MpNEK1^dKD^ (KD deletion) were planted in the medium with (+ER) or without 1 µM estradiol (Mock) and grown for 14 days. (c) The gemmae of the WT and inducible lines were grown as in (b) for 7 days.

To determine whether the kinase activity of MpNEK1 is required for growth suppression, we generated the inducible lines of MpNEK1 without its kinase activity (Fig. 9). For this purpose, lysine-34 essential for the kinase activity was substituted with glutamic acid (K37E, MpNEK1^K37E^) or the N-terminal kinase domain was deleted (KD deletion, MpNEK1^dKD^) (Fig. 9a). MpNEK1^K37E^ and MpNEK1^dKD^ were fused with Citrine at the C-termini and constructed into the XVE system as shown in Fig. 9a.

**Figure 10.**
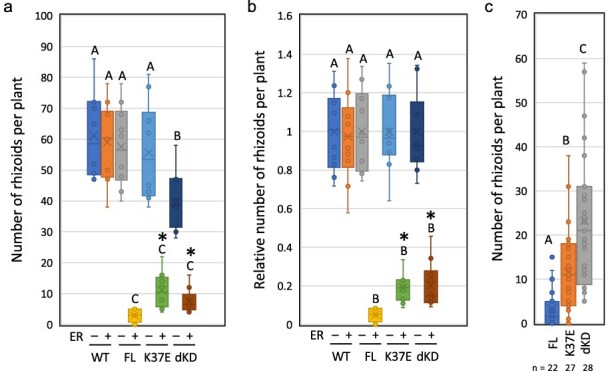
Effect of MpNEK1 induction on the number of rhizoids. (a) The number of rhizoids in the gemmae grown for 7 days in the medium with (+ER) or without 1 µM estradiol (Mock). The gemmae of the WT and inducible lines of full-length MpNEK1–Citrine (FL), MpNEK1^K37E^–Citrine (K37E), and MpNEK1^dKD^–Citrine (dKD) were used. Data are shown by box plots (*n* = 10 plants). The different letters indicate significant differences by Tukey’s Honest Significant Difference (HSD) test (*P* < .03). Asterisks indicate significant differences from the value of FL with estradiol (+ER) (*t*-test, *P* < .01). (b) The relative number of rhizoids in (a). The average number of rhizoids in estradiol-free media (−ER) in each genotype was used for the normalization. Data are shown by box plots (*n* = 10 plants). The different letters indicate significant differences by Tukey’s HSD test (*P* < .03). Asterisks indicate significant differences from the value of FL with estradiol (+ER) (*t*-test, *P* < .01). (c) The number of rhizoids in the gemmae grown for 14 days in the medium supplemented with 1 µM estradiol. The gemmae of the inducible lines of full-length MpNEK1–Citrine (FL), MpNEK1^K37E^–Citrine (K37E), and MpNEK1^dKD^–Citrine (dKD) were used. Data are shown by box plots (*n* indicates the number of plants). The different letters indicate significant differences by Tukey’s HSD test (*P* < .03).

We compared the effects of MpNEK1^K37E^ and MpNEK1^dKD^ induction to that of the full-length MpNEK1 induction (Figs 9 and 10, Supplementary Fig. S11). The gemmae of the WT and inducible lines of full-length MpNEK1, MpNEK1^K37E^, and MpNEK1^dKD^ were grown in the medium with (+ER) or without 1 µM estradiol (Mock). In the presence of estradiol, thallus growth was suppressed in all inducible lines (Fig. 9b). This finding indicates that the kinase activity of MpNEK1 is not required for the suppression of thallus growth. However, close-up observation showed that the thalli of MpNEK1^K37E^- and MpNEK1^dKD^-inducible lines were larger than that of full-length MpNEK1-inducible line (Fig. 9c). Furthermore, rhizoids were scarcely formed in the full-length inducible lines, whereas MpNEK1^K37E^- and MpNEK1^dKD^-inducible lines developed several rhizoids, which elongated as those of the WT (Fig. 9c).

We quantified the number of rhizoids, which is a good indicator of the difference among the inducible lines (Fig. 10). The gemmae of the WT and inducible lines of full-length MpNEK1, MpNEK1^K37E^, and MpNEK1^dKD^ were grown in the medium with (+ER) or without 1 µM estradiol (Mock) for 7 days (Fig. 10a). The number of rhizoids in the WT was not affected by estradiol. Although the number of rhizoids was significantly decreased in all of the inducible lines, the inducible lines of MpNEK1^K37E^ and MpNEK1^dKD^ developed more rhizoids than the full-length inducible lines. This difference was confirmed in the normalized data by using the average number of rhizoids in the estradiol-free medium as a standard (Fig. 10b). The difference between kinase-deficient and full-length inducible lines was more clear in the quantification of rhizoids in 14-day treatment (Fig. 10c). Thus, the induction of kinase-deficient MpNEK1 is slightly milder than the full-length induction. The kinase activity of MpNEK1 may have a little contribution to growth suppression.

**Figure 11.**
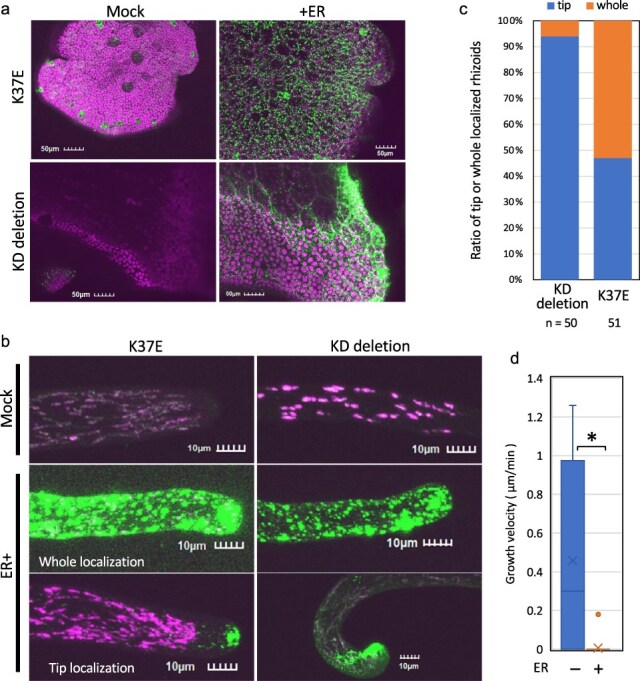
Localization of kinase-deficient MpNEK1–Citrine. (a) The gemmae of the inducible lines of MpNEK1^K37E^–Citrine (K37E) or MpNEK1^dKD^–Citrine (KD deletion) were planted in the medium with (+ER) or without 1 µM estradiol (Mock), grown for 4 days, and observed under a confocal microscope. Green: MpNEK1–Citrine, magenta: plastid autofluorescence. (b) The gemmae of the inducible lines of MpNEK1^K37E^–Citrine (K37E) or MpNEK1^dKD^–Citrine (KD deletion) were grown as in (a) and rhizoids were observed under a confocal microscope. Green: MpNEK1–Citrine, magenta: plastid autofluorescence. (c) Ratio of rhizoids with tip-localized or whole localized MpNEK1^dKD^–Citrine (KD deletion) or MpNEK1^K37E^–Citrine (K37E) after 120 hours of estradiol treatment. (d) Growth velocity of rhizoids in the MpNEK1^K37E^–Citrine-inducible lines incubated for 48 hours with (+ER) or without 1 µM estradiol (−ER). Data are shown by box plots (*n* = 30 rhizoids). An asterisk indicates significant difference (*t*-test, *P* < .01).

To investigate the accumulation and subcellular localization of kinase-deficient MpNEK1, inducible lines were grown in the medium with or without 1 µM estradiol and observed under confocal microscopy (Fig. 11). Because the inducible lines of kinase-deficient MpNEK1 develop larger thalli and more rhizoids than the full-length inducible lines, they provide good opportunity to analyze localization of MpNEK1 in the growing organs and more viable cells. Estradiol treatment induced accumulation of MpNEK1^K37E^ and MpNEK1^dKD^ in the granules throughout the thallus as the full-length MpNEK1 (Fig. 11a).

We observed localization of MpNEK1^K37E^ and MpNEK1^dKD^ in rhizoids (Fig. 11b). Some rhizoids showed granular accumulation throughout rhizoids (whole localization), while others showed preferential localization at the apex of rhizoids (tip localization). We quantified the number of rhizoids with the whole localization or tip localization (Fig. 11c). Tip-localized rhizoids were ∼47% in MpNEK1^K37E^ while 94% in MpNEK1^dKD^. Thus, MpNEK1^dKD^ exhibited relatively normal localization compared with MpNEK1^K37E^ in the inducible condition.

**Figure 12.**
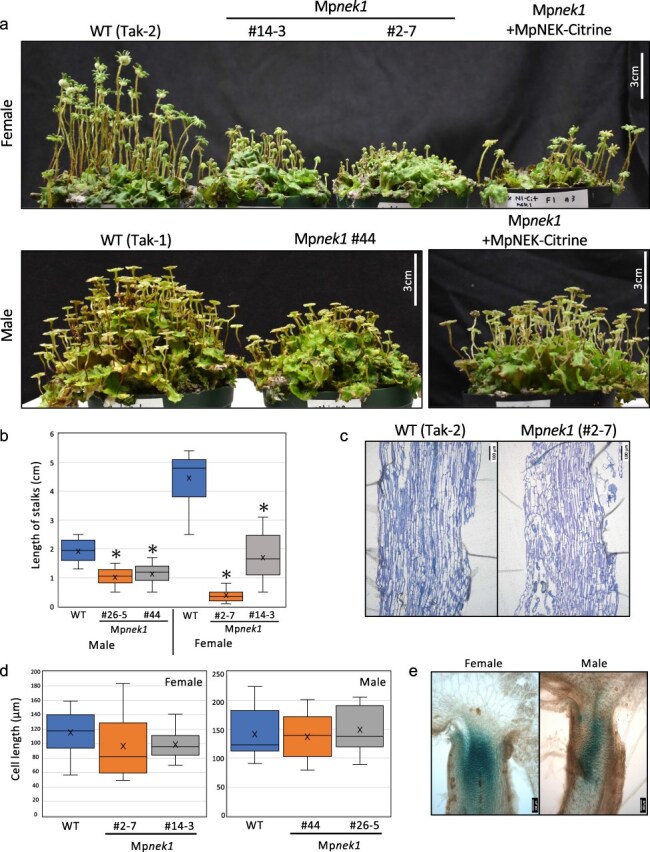
Growth suppression of gametangiophores in the Mp*nek1* mutants. (a) Overall morphology of archegoniophores (upper panel) and antheridiophores (lower panels) of 2-month-old plants. (b) Quantification of the length of stalks of gametangiophores. Asterisks indicate significant differences from WT (*t*-test, P < 0.05, n = 20). (c) Longitudinal sections of stalks of archegoniophores of the WT and Mp*nek1* mutant. (d) Quantification of the length of stalk cells of gametangiophores (n = 20 in female, 10 in male). (e) Promoter activity of Mp*NEK1* in the stalks of gametangiophores.

Next, we measured the velocity of rhizoid growth in the MpNEK1^K37E^-inducible lines (Fig. 11d). Rhizoid growth was almost stopped after 48 hours of estradiol treatment. This is comparable to the effect of the full-length inducible lines. Therefore, the excess accumulation of kinase-dead MpNEK1 also suppressed rhizoid elongation.

### Mp*nek1* mutants exhibit growth suppression of reproductive organs

Our gain-of-function approach suggests the involvement of MpNEK1 in cell division and proliferation. To verify this, we analyzed the phenotype of Mp*nek1* mutants (Fig. 12). Because Mp*nek1* mutant thalli showed a relatively mild phenotype, wavy morphology, and the same size as WT thalli (Otani et al. 2018), it is difficult to estimate the function of MpNEK1 in cell division and meristematic activity in thalli. Hence, we monitored the growth of reproductive organs, gametangiophores. Mp*nek1* mutants showed obvious growth suppression of gametangiophores in both female and male plants (Fig. 12a and b). To determine whether this growth suppression is due to reduced cell elongation, we analyzed cell length in stalks (Fig. 12c and d). The length of stalk cells in Mp*nek1* was almost identical to that in the WT. These results suggest that growth suppression of gametangiophores is attributed to the reduced cell division. This was supported by the preferential activity of Mp*NEK1* promoter in the upper region of the stalks (Fig. 12e), which is the meristematic zone of stalks.

## Discussion

Here, we established and characterized the induction system of MpNEK1 as a new tool to analyze plant NEK function. Estradiol-based XVE induction system has been widely used in angiosperms, but its application and characterization in *M. polymorpha* still remain limited. The efficient induction of Mp*NEK1* transcripts and overaccumulation of MpNEK1–Citrine fusion protein throughout the plant indicate that this system is very helpful for the functional analysis of MpNEK1 and potentially for those of other proteins in *M. polymorpha*.

We noticed several unexpected phenomena in which caution is required. MpNEK1–Citrine showed leaky expression in the meristem without estradiol. This might be due to the basal expression of artificial transcriptional activator XVE under the control of Mp*EF1α* promoter, which is highly active in meristematic tissues (Althoff et al. 2014). Nonetheless, this leaky expression did not affect thallus growth, suggesting that the effective threshold of NEK may exist. Since the leaky expression varied in plants and transgenic lines, estrogen-free XVE-mediated transactivation might be stochastic and/or responsive to the fluctuations of physiological states.

Second, estrogen-resistant silenced plants emerged as survivors in the prolonged treatment, especially at the lower concentrations of estradiol. This resistant phenotype could be transferred to the next-generation gemmae (e.g. DNA methylation, siRNA movement). Thus, it would be useful for the analysis of gene silencing in *M. polymorpha*, while invoking concerns about treatment time and estradiol concentration to minimize the contamination of silenced plants.

In summary, our results show that the short-term treatment (∼24 to 48 hours) with relatively higher concentrations of estradiol (1–10 µM) is suitable to avoid the silencing effect. Furthermore, leaky expression does not affect plant growth in our experimental condition. Nevertheless, an alternative inducible system with dexamethasone or heat treatment would be more beneficial to avoid these issues. Dexamethasone-inducible translocation system has been successfully utilized for transcriptional regulators (Yamaoka et al. 2018, Yasui et al. 2019), while a transactivation system is required for the analysis of non-nuclear proteins. Dexamethasone-inducible transactivation system (e.g. Gal4/GVG, pOp6/LhGR) is not applied in *M. polymorpha* and has been reported to suppress plant growth in several species (e.g. Kang et al. 1999). The heat-inducible system is also useful for gene induction and conditional knockout (Nishihama et al. 2016), while its use for gene/protein induction may not be suitable for thermolabile proteins and heat-sensitive phenotypes (e.g. Furumoto et al. 2024). Although further improvement will be required by referring to the optimization of the XVE system in a moss *Physcomitrium patens* (Kubo et al. 2013) and other systems, the estradiol-inducible system is very informative for functional analysis because of its less toxic effect and high induction efficiency.

MpNEK1 induction severely suppressed thallus growth probably through cell proliferation. There are several possible mechanisms of this growth-suppressing effect. First, MpNEK1 may depolymerize microtubule-based mitotic apparatus including spindle and phragmoplast or affect cortical microtubule organization, which is required for directional growth prior to cell division. However, our microtubule imaging showed that overexpression of MpNEK1 did not severely affect the mitotic apparatus. During the interphase, MpNEK1 reduced the density and dynamics of cortical microtubules but did not severely affect cell growth and morphology. MpNEK1 may depolymerize cortical microtubules but its effect is under the threshold and/or masked by other regulators. Second, overexpression of MpNEK1 may activate cell cycle checkpoints to prevent cell cycle progression. According to the experiment of EdU labeling, the cells through the S phase were reduced under the MpNEK1 overexpression. Furthermore, MpNEK1 induction reduced mitotic cells in the M phase. Thus, MpNEK1 induction may stop the cell cycle in the G1 phase or G1/S transition to eventually reduce the number of dividing cells. The *nimA* mutants of a filamentous fungus *Aspergillus nidulans* exhibit cell cycle arrest just before the M phase, and conversely, overexpression of *NimA* causes premature chromosome condensation and aberrant spindle formation, resulting in cell cycle arrest (Oakley and Morris 1983, Osmani et al. 1988). Taking into account of remarkable promoter activity of Mp*NEK1* in the meristem (Otani et al. 2018), it would be likely that MpNEK1 controls cell cycle progression and checkpoints.

Overexpression of MpNEK1 suppressed tip growth in rhizoids. Furthermore, overexpression altered localization of MpNEK1–Citrine from the rhizoid apex to the entire region as the time of estradiol treatment increased. This may reflect the overflow of MpNEK1 from the transport and localization machineries. As a consequence, tip growth was stopped in rhizoids with this dispersed localization pattern. Over-accumulated MpNEK1 may affect microtubule organization and/or microtubule-dependent processes during rhizoid growth. Because the longitudinal microtubules promote rhizoid elongation through organelle transport (Kanda et al. 2022), it is plausible that MpNEK1 accumulation throughout rhizoids suppresses the transport system (see the last paragraph).

Unexpectedly, we found growth suppression of thalli and rhizoids by kinase-deficient MpNEK1. This result demonstrates that growth suppression is mainly independent of protein phosphorylation by MpNEK1. Therefore, the induction system is not suitable for the identification of target proteins phosphorylated by MpNEK1. Nevertheless, kinase-dead MpNEK1 is slightly milder than the WT MpNEK1 in the estradiol induction system. The kinase activity of MpNEK1 is not essential for growth suppression but may have a minor contribution. The most plausible scenario is that excessively accumulated MpNEK1 might trap interacting proteins and substrates in the granules to suppress thallus growth and rhizoid elongation in a kinase-independent manner. This is supported by the findings that the C-terminal regulatory regions of plant NEKs interact with various proteins (Pnueli et al. 2001, Sakai et al. 2008, Motose et al. 2011). Animal NEK7 licenses the assembly and activation of NLRP3 inflammasome, a large protein complex for the activation of inflammatory caspases, independently of its kinase activity (He et al. 2016, Sharif et al. 2019). Furthermore, NEK7 is required for the oligomerization of NLRP3 and its adaptor protein ASC, which forms the granular structure in the cytosol (He et al. 2016, Sharif et al. 2019). Plant NEK may also function as the assembly factor of protein supercomplex and cytosolic granular structures.

In summary, the MpNEK1 induction system is useful for the analyses of its function in cell division and proliferation. Furthermore, it would be helpful to visualize directional protein transport in rhizoids because we observed MpNEK1 particles moved to the acropetal and basipetal direction (Videos S3–S5). The velocity of MpNEK1 particles is quite similar to that of armadillo-repeat kinesin (Kanda et al. 2022), about half of velocity of growing microtubule plus ends, suggesting this motility is not due to plus-end tracking but rather to the kinesin-dependent transport along microtubules. MpNEK1 overexpression has the potent growth effect than that of *Arabidopsis* NEK6, whose overexpression caused a slight reduction of root growth (Takatani et al. 2017). Compared with MpNEK1, NEK6–Citrine fusion protein is not highly accumulated in the XVE system (H.Mo., unpublished result), implying the differential degradation efficiency of NEK proteins and the potential advantage of protein induction and analyses in *M. polymorpha*.

## Materials and Methods

### Plant materials and growth conditions

*M. polymorpha* accessions Takaragaike-1 (Tak-1, male) and Takaragaike-2 (Tak-2, female) were used as the WT strains. Mp*nek1* mutants and Mp*NEK1pro:GUS* lines were reported in Otani et al. (2018). Plants were grown on the half-strength Gamborg’s B5 medium solidified with 1% agar at 22°C under the light cycles with 16 hours of light and 8 hours of darkness.

### Plasmid construction and transformation

Plasmids were mainly constructed based on the Gateway Technology (Life technologies). Primers used in the plasmid construction are listed in Table S1. To generate a plasmid of Mp*NEK1* induction by estradiol, the full-length MpNEK1 coding sequence in the pENTR/D-TOPO entry vector (Otani et al. 2018) was transferred by LR reaction (LR clonase II enzyme mix, Life Technologies) into the Gateway binary vector pMpGWB344 (MpEF1α pro:XVE≫LexA operator:Gateway cassette).

To generate a plasmid of estradiol-inducible MpNEK1-mCitrine, *mCitrine* coding sequence was amplified by KOD One (Toyobo) and a primer set (Table S1) and was cloned into the *Asc*I site of the pENTR/D-TOPO entry vector containing full-length MpNEK1 CDS (Otani et al. 2018) by In-fusion system (Takara). The resulting vector pENTR/D–TOPO–MpNEK1–mCitrine was subjected to LR reaction to transfer MpNEK1–Citrine fusion into the Gateway binary vector pMpGWB144 (MpEF1α pro:XVE≫LexA operator:Gateway cassette).

To generate a plasmid of estradiol-inducible kinase-deficient MpNEK1, pENTR/D–TOPO–MpNEK1–mCitrine was subjected to the inverse PCR-based mutagenesis using KOD mutagenesis kit (Toyobo) and primer sets (Table S1). The resulting vectors, pENTR/D–TOPO–MpNEK1^K37E^–mCitrine and pENTR/D–TOPO–MpNEK1^dKD^–mCitrine were subjected to LR reaction to transfer mutated MpNEK1–Citrine fusion into the Gateway binary vector pMpGWB144 (MpEF1α pro:XVE≫LexA operator:Gateway cassette).

The constructs, pMpGWB344–MpNEK1 (MpEF1α pro:XVE≫LexA operator:MpNEK1), pMpGWB144–MpNEK1–Citrine (MpEF1α pro:XVE≫LexA operator:MpNEK1–Citrine), pMpGWB144–MpNEK1^K37E^–Citrine (MpEF1α pro:XVE≫LexA operator:MpNEK1^K37E^–Citrine), and pMpGWB144–MpNEK1^dKD^–Citrine (MpEF1α pro:XVE≫LexA operator:MpNEK1^dKD^–Citrine) were transformed into F1 sporelings derived from sexual crosses between Tak-2 and Tak-1 by *Agrobacterium*-mediated method (Ishizaki et al. 2008). The pMpGWB344–MpNEK1 construct was transformed into the regenerating thalli of CaMV35Spro:Citrine–MpTUB2 plants harboring pMpGWB105–MpTUB2 construct (Otani et al. 2018) according to the method of Kubota et al. (2013). Chlorsulfuron and hygromycin were used for the selection of transformants in pMpGWB344 and pMpGWB144, respectively. In pMpGWB344 and pMpGWB144, the artificial transcriptional activator XVE is expressed under the control of Mp*EF1α* promoter. XVE translocates to the nucleus in the presence of estradiol and binds to the LexA operator to induce the expression of MpNEK1– and MpNEK1–Citrine, respectively.

### Quantitative real-time PCR

Total RNA was isolated from the thalli of the WT and transgenic plants. For each sample, 0.5 µg of total RNA was reverse transcribed to cDNA using ReverTra Ace reverse transcriptase (Toyobo) according to the manufacturer’s protocol. Real-time PCR was performed on a thermal cycler Dice Real Time System (Takara) using KOD SYBR qPCR Kit (Toyobo) according to the manufacturer’s protocol. Transcript levels of Mp*EF1α* and Mp*ACT* were used as a reference for normalization (Saint-Marcoux et al. 2015). Primers used in RT-qPCR are listed in Table S1. RT-qPCR experiments were performed using four biological replicates.

### Microscopy

The morphology of gemmalings and rhizoids was observed under a stereo microscope S8APO0 (Leica Microsystems) or a light microscope DM5000B (Leica) equipped with a Charge Coupled Device (CCD) camera DFC500 (Leica). The morphology of gametangiophores was photographed by a single-lens reflex camera D5600 (Nikon, Tokyo Japan). Beta-glucuronidase (GUS) staining was conducted according to Otani et al. (2018) and observed under DM5000B equipped with DFC500. To analyze the localization of MpNEK1–Citrine, plants were grown on half-strength B5 medium for 3 and 5 days under cycles with 16 hours of light and 8 hours of darkness. For live imaging, half-strength B5 agar medium was solidified in the glass bottom dish (35 mm in diameter × 10 mm in thickness, Thickness No. 1, Matsunami) and then the central region of agar medium on the bottom glass strip was removed by tweezers. This region was poured with ∼200 µl of melted half-strength B5 agar medium to generate a thin solidified medium. The gemmae were planted and grown in the central thin medium for 2–7 days. The details of this method will be described elsewhere by H.M. Gemmalings were observed under a FV1200 confocal laser-scanning microscope (Olympus) equipped with a high-sensitivity GaAsP detector and silicone oil objective lenses (30×, 60× Olympus) or FV3000 (Olympus) equipped with high-sensitivity GaAsP detectors and oil immersion objective lenses (40×, NA = 1.4; 60×, NA = 1.42, Olympus) or water immersion objective lenses (60×, NA = 1.2, Olympus). Silicone oil (SIL300CS, Olympus), immersion oil (F30CC, Olympus), or deionized water was used as immersion media for these objective lenses. The samples were excited at 473 and 559 nm (laser diode) and the emission was separated using a FV12-MHSY SDM560 filter (490–540 and 575–675 nm, Olympus) in FV1200. The samples were excited at 488 and 561 nm (laser diode) and the emission was separated using the multichannel TruSpectral detection (500–540 and 600–700 nm, Olympus) in FV3000. The images were analyzed using ImageJ (National Institutes of Health, USA) and LPIXEL ImageJ Plugins (https://lpixel.net/products/lpixel-imagej-plugins/). Microtubule bundling (skewness) and density (occupancy) were determined according to Higaki et al. (2010). Microtubule dynamics was quantified according to Abe et al. (2005). For estradiol treatment, an aliquot (200–300 µl) of 1/2 B5 liquid medium supplemented with estradiol at a concentration of 1 µM or 10 µM was added to the gemmalings for the induction of Mp*NEK1*. As a control, the same amount of 1/2 B5 liquid medium supplemented with the same concentration of solvent (dimethyl sulfoxide, <0.1%) was applied.

### EdU staining

S-phase cells were visualized using Click-iT EdU Imaging Kits (Life Technologies) according to the manufacturer’s instructions. Gemmae were incubated on the half-strength Gamborg’s B5 agar medium supplemented with or without 1 µM β-estradiol for 4 days. Alternatively, for 1-day treatment, gemmae were incubated on the agar medium without 1 µM β-estradiol for 3 days and then transferred to and incubated in the agar medium with 1 µM β-estradiol for 1 day. Gemmalings were then transferred to a solution containing 10 µM EdU and incubated for 1 hour. Plants were fixed with 3.7% formaldehyde solution in phosphate-buffered saline for 20 minutes. EdU incorporated into DNA was labeled with Alexa Fluor 488-azide-containing Click-iT reaction cocktail in the dark for 30 minutes. EdU-labeled cells were observed using a confocal laser scanning microscope, FV1200 (Olympus). The maximum Z-projection images were created using the ImageJ software.

## Supplementary Material

pcaf021_Supp

## Data Availability

The data underlying this article will be shared on reasonable request to the corresponding author.

## References

[R1] Abe T. and Hashimoto T. (2005) Altered microtubule dynamics by expression of modified alpha-tubulin protein causes right-handed helical growth in transgenic Arabidopsis plants. *Plant J*. 43: 191–204. 15998306 10.1111/j.1365-313X.2005.02442.x

[R2] Althoff F., Kopischke S., Zobell O., Ide K., Ishizaki K., Kohchi T., et al. (2014) Comparison of the Mp*EF1α* and *CaMV35* promoters for application in *Marchantia polymorpha* overexpression studies. *Transgenic Res*. 23: 235–244. 24036909 10.1007/s11248-013-9746-z

[R3] Bao H., Sun R., Iwano M., Yoshitake Y., Aki S.S., Umeda M., et al. (2024) Conserved CKI1-mediated signaling is required for female germline specification in *Marchantia polymorpha*. *Curr. Biol*. 34: 1324–1332. 38295795 10.1016/j.cub.2024.01.013

[R4] Bowman J.L., Kohchi T., Yamato K.T., Jenkins J., Shu S., Ishizaki K., et al. (2017) Insights into land plant evolution garnered from the *Marchantia polymorpha* genome. *Cell* 171: 287–304. 28985561 10.1016/j.cell.2017.09.030

[R5] Buschmann H., Holtmannspötter M., Borchers A., O’Donoghue M.T. and Zachgo S. (2016) Microtubule dynamics of the centrosome-like polar organizers from the basal land plant *Marchantia polymorpha*. *New Phytol*. 209: 999–1013. 26467050 10.1111/nph.13691

[R6] Champion C., Lamers J., Jones V.A.S., Morieri G., Honkanen S. and Dolan L. (2021) Microtubule associated protein WAVE DAMPENED2-LIKE (WDL) controls microtubule bundling and the stability of the site of tip-growth in *Marchantia polymorpha* rhizoids. *PLoS Genet*. 17: e1009533. 10.1371/journal.pgen.1009533 PMC8177534 34086675

[R7] Flores-Sandoval E., Dierschke T., Fisher T.J. and Bowman J.L. (2016) Efficient and inducible use of artificial microRNAs in *Marchantia polymorpha*. *Plant Cell Physiol*. 57: 281–290. 25971256 10.1093/pcp/pcv068

[R8] Fry A.M., O’Regan L., Sabir S.R. and Bayliss R. (2012) Cell cycle regulation by the NEK family of protein kinases. *J. Cell Sci*. 125: 4423–4433. 23132929 10.1242/jcs.111195 PMC3500863

[R9] Furumoto T., Yamaoka S., Kohchi T., Motose H. and Takahashi T. (2024) Thermospermine is an evolutionarily ancestral phytohormone required for organ development and stress responses in *Marchantia polymorpha*. *Plant Cell Physiol*. 65: 460–471. 38179828 10.1093/pcp/pcae002 PMC11020214

[R10] Furuya T., Nishihama R., Ishizaki K., Kohchi T., Fukuda H. and Kondo Y. (2022) A glycogen synthase kinase 3-like kinase MpGSK regulates cell differentiation in *Marchantia polymorpha*. *Plant Biotech*. 39: 65–72. 10.5511/plantbiotechnology.21.1219a PMC9200085 35800965

[R11] He Y., Zeng M.Y., Yang D., Motro B. and Núñez G. (2016) NEK7 is an essential mediator of NLRP3 activation downstream of potassium efflux. *Nature* 530: 354–359. 26814970 10.1038/nature16959 PMC4810788

[R12] Higaki T., Kutsuna N., Sano T., Kondo N. and Hasezawa S. (2010) Quantification and cluster analysis of actin cytoskeletal structures in plant cells: role of actin bundling in stomatal movement during diurnal cycles in Arabidopsis guard cells. *Plant J*. 61: 156–165. 20092030 10.1111/j.1365-313x.2009.04032.x

[R13] Honkanen S., Jones V.A.S., Morieri G., Champion C., Hetherington A.J., Kelly S., et al. (2016) The mechanism forming the cell surface of tip-growing rooting cells is conserved among land plants. *Curr. Biol*. 26: 3238–3244. 27866889 10.1016/j.cub.2016.09.062 PMC5154754

[R14] Ishizaki K., Chiyoda S., Yamato K.T. and Kohchi T. (2008) *Agrobacterium*-mediated transformation of the haploid liverwort *Marchantia polymorpha* L., an emerging model for plant biology. *Plant Cell Physiol*. 49: 1084–1091. 18535011 10.1093/pcp/pcn085

[R15] Ishizaki K., Nishihama R., Ueda M., Inoue K., Ishida S., Nishimura Y., et al. (2015) Development of gateway binary vector series with four different selection markers for the liverwort *Marchantia polymorpha*. *PLoS One* 10: e0138876. 10.1371/journal.pone.0138876 PMC4583185 26406247

[R16] Ishizaki K., Nishihama R., Yamato K.T. and Kohchi T. (2016) Molecular genetic tools and techniques for *Marchantia polymorpha* research. *Plant Cell Physiol*. 57: 262–270. 26116421 10.1093/pcp/pcv097

[R17] Jones V.A.S. and Dolan L. (2012) The evolution of root hairs and rhizoids. *Annal. Bot*. 110: 205–212. 10.1093/aob/mcs136 PMC3394659 22730024

[R18] Kanda A., Otani K., Takahashi T. and Motose H. (2022) Plant specific armadillo repeat kinesin directs organelle transport and microtubule convergence to promote tip growth. *BioRxiv*. doi: 10.1101/2022.07.08.499237

[R19] Kang H.G., Fang Y. and Singh K.B. (1999) A glucocorticoid-inducible transcription system causes severe growth defects in Arabidopsis and induces defense-related genes. *Plant J*. 20: 127–133. 10571872 10.1046/j.1365-313x.1999.00575.x

[R20] Kohchi T., Yamato K.T., Ishizaki K., Yamaoka S. and Nishihama R. (2021) Development and molecular genetics of *Marchantia polymorpha*. *Ann. Rev. Plant Biol*. 72: 677–702. 33684298 10.1146/annurev-arplant-082520-094256

[R21] Kubo M., Imai A., Nishiyama T., Ishikawa M., Sato Y., Kurata T., et al. (2013) System for stable beta-estradiol-inducible gene expression in the moss *Physcomitrella patens*. *PLoS One* 8: e77356. 10.1371/journal.pone.0077356 PMC3785464 24086772

[R22] Kubota A., Ishizaki K., Hosaka M. and Kohchi T. (2013) Efficient *Agrobacterium*-mediated transformation of the liverwort *Marchantia polymorpha* using regenerating thalli. *Biosci. Biotechnol. Biochem*. 77: 167–172. 23291762 10.1271/bbb.120700

[R23] Mase H., Nakagami H., Okamoto T., Takahashi T. and Motose H. (2022) Establishment and application of novel culture methods in *Marchantia polymorpha*: persistent tip growth is required for substrate penetration by rhizoids. *Commun. Integr. Biol*. 15: 164–167. 35832537 10.1080/19420889.2022.2095137 PMC9272829

[R24] Motose H., Hamada T., Yoshimoto K., Murata T., Hasebe M., Watanabe Y., et al. (2011) NIMA-related kinases 6, 4, and 5 interact with each other to regulate microtubule organization during epidermal cell expansion in *Arabidopsis thaliana*. *Plant J*. 67: 993–1005. 21605211 10.1111/j.1365-313X.2011.04652.x

[R25] Motose H., Tominaga R., Wada T., Sugiyama M. and Watanabe Y. (2008) A NIMA-related protein kinase suppresses ectopic outgrowth of epidermal cells through its kinase activity and the association with microtubules. *Plant J*. 54: 829–844. 18266916 10.1111/j.1365-313X.2008.03445.x

[R26] Nishihama R., Ishida S., Urawa H., Kamei Y. and Kohchi T. (2016) Conditional gene expression/deletion systems for *Marchantia polymorpha* using its own heat-shock promoter and Cre/loxP-mediated site-specific recombination. *Plant Cell Physiol*. 57: 271–280. 26148498 10.1093/pcp/pcv102

[R27] Oakley B.R. and Morris N.R. (1983) A mutation in *Aspergillus nidulans* that blocks the transition from interphase to prophase. *J. Cell Biol*. 96: 1155–1158. 6339527 10.1083/jcb.96.4.1155 PMC2112314

[R28] O’Connell M.J., Krien M.J. and Hunter T. (2003) Never say never. The NIMA-related protein kinases in mitotic control. *Trends Cell Biol*. 13: 221–228. 12742165 10.1016/s0962-8924(03)00056-4

[R29] Osmani S.A., Pu R.T. and Morris N.R. (1988) Mitotic induction and maintenance by overexpression of a G2-specific gene that encodes a potential protein kinase. *Cell* 53: 237–244. 3359487 10.1016/0092-8674(88)90385-6

[R30] Otani K., Ishizaki K., Nishihama R., Takatani S., Kohchi T., Takahashi T., et al. (2018) An evolutionarily conserved NIMA-related kinase directs rhizoid tip growth in the basal land plant *Marchantia polymorpha*. *Development* 145: dev154617. 10.1242/dev.154617 29440300

[R31] Pnueli L., Gutfinger T., Hareven D., Ben-Naim O., Ron N., Adir N., et al. (2001) Tomato SP-interacting proteins define a conserved signaling system that regulates shoot architecture and flowering. *Plant Cell* 13: 2687–2702. 11752381 10.1105/tpc.010293 PMC139482

[R32] Saint-Marcoux D., Proust H., Dolan L., Langdale J.A. and Margis R. (2015) Identification of reference genes for real-time quantitative PCR experiments in the liverwort *Marchantia polymorpha*. *PLoS One* 10: e0118678. 10.1371/journal.pone.0118678 PMC4370483 25798897

[R33] Sakai T., Honing H., Nishioka M., Uehara Y., Takahashi M., Fujisawa N., et al. (2008) Armadillo repeat-containing kinesins and a NIMA-related kinase are required for epidermal-cell morphogenesis in *Arabidopsis*. *Plant J*. 53: 157–171. 17971038 10.1111/j.1365-313X.2007.03327.x

[R34] Sharif H., Wang L., Wang W.L., Magupalli V.G., Andreeva L., Qiao Q., et al. (2019) Structural mechanism for NEK7-licensed activation of NLRP3 inflammasome. *Nature* 570: 338–347. 31189953 10.1038/s41586-019-1295-z PMC6774351

[R35] Shimamura M. (2016) *Marchantia polymorpha*: taxonomy, phylogeny and morphology of a model system. *Plant Cell Physiol*. 57: 230–256. 26657892 10.1093/pcp/pcv192

[R36] Takatani S., Otani K., Kanazawa M., Takahashi T. and Motose H. (2015) Structure, function, and evolution of plant NIMA-related kinases: implication for phosphorylation-dependent microtubule regulation. *J. Plant Res*. 128: 875–891. 26354760 10.1007/s10265-015-0751-6

[R37] Takatani S., Ozawa S., Yagi N., Hotta T., Hashimoto T., Takahashi Y., et al. (2017) Directional cell expansion requires NIMA-related kinase 6 (NEK6)-mediated cortical microtubule destabilization. *Sci. Rep*. 7: 7826. 10.1038/s41598-017-08453-5 PMC5552743 28798328

[R38] Takatani S., Verger S., Okamoto T., Takahashi T., Hamant O. and Motose H. (2020) Microtubule response to tensile stress is curbed by NEK6 to buffer growth variation in the *Arabidopsis* hypocotyl. *Curr. Biol*. 30: 1491–1503. 32169210 10.1016/j.cub.2020.02.024

[R39] Yamaoka S., Nishihama R., Yoshitake Y., Ishida S., Inoue K., Saito M., et al. (2018) Generative cell specification requires transcription factors evolutionarily conserved in land plants. *Curr. Biol*. 28: 479–486. 29395928 10.1016/j.cub.2017.12.053

[R40] Yasui Y., Tsukamoto S., Sugaya T., Nishihama R., Wang Q., Kato H., et al. (2019) GEMMA CUP-ASSOCIATED MYB1, an ortholog of axillary meristem regulators, is essential in vegetative reproduction in *Marchantia polymorpha*. *Curr. Biol*. 29: 3987–3995. 31708390 10.1016/j.cub.2019.10.004

[R41] Zuo J., Niu Q.W. and Chua N.H. (2000) An estrogen receptor-based transactivator XVE mediates highly inducible gene expression in transgenic plants. *Plant J*. 24: 265–273. 11069700 10.1046/j.1365-313x.2000.00868.x

