# Supplementary material for: Involvement of a NIMA-related kinase in cell division of the liverwort Marchantia polymorpha
Source: Plant Cell Physiol. 2025 Feb 17;66(5):815–32. doi: 10.1093/pcp/pcaf021 (PMC12125575; doi:10.1093/pcp/pcaf021)
Supplement: pcaf021_Supp [file pcaf021_supp.zip › suppl_data/pcp-2024-e-00281-File014.pdf]

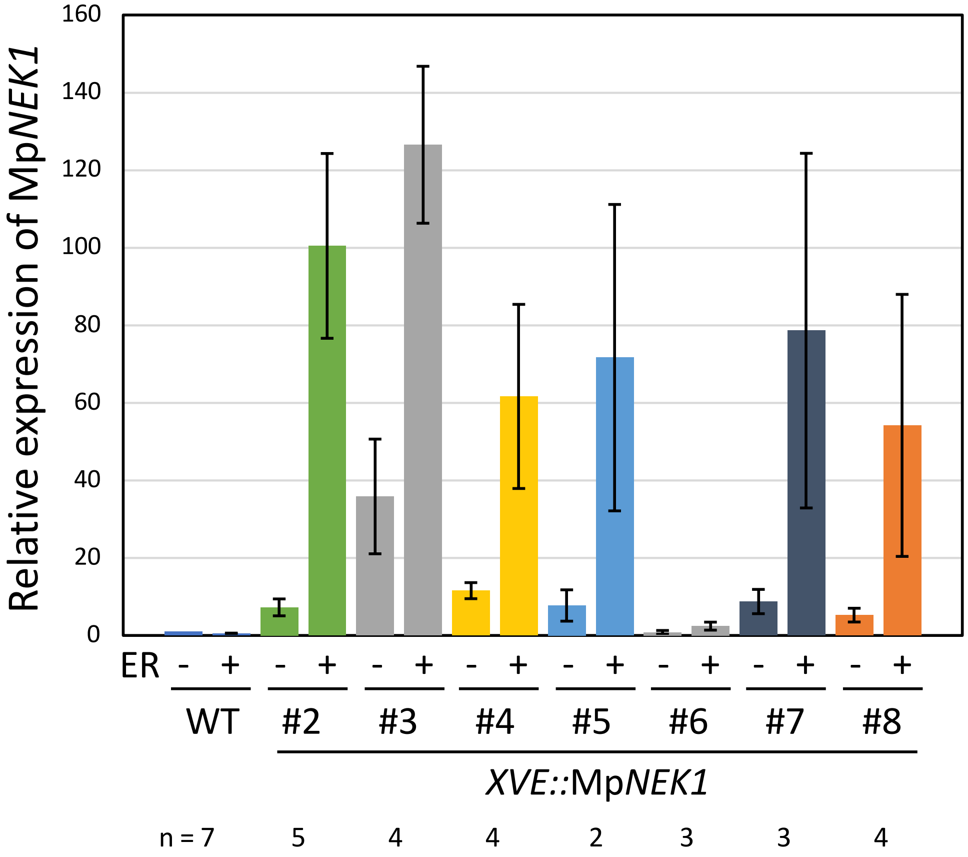

Fig S1. Effects of estradiol on the accumulation of Mp*NEK1* transcripts in the wild type and transgenic lines introduced with *XVE::*Mp*NEK1*.

The 10-day old plants of the wild type (WT) and the transgenic lines were incubated for 1 day in the medium with (+) or without 10 µM estradiol (-). Total RNA was extracted and subjected to RT-qPCR. All transcript levels are relative to that of mock-treated wild type plants. Mp*EF1α* and Mp*ACT* were used as the control genes. Columns and error bars indicate mean values and standard errors, respectively (n indicates the number of biological replicates).

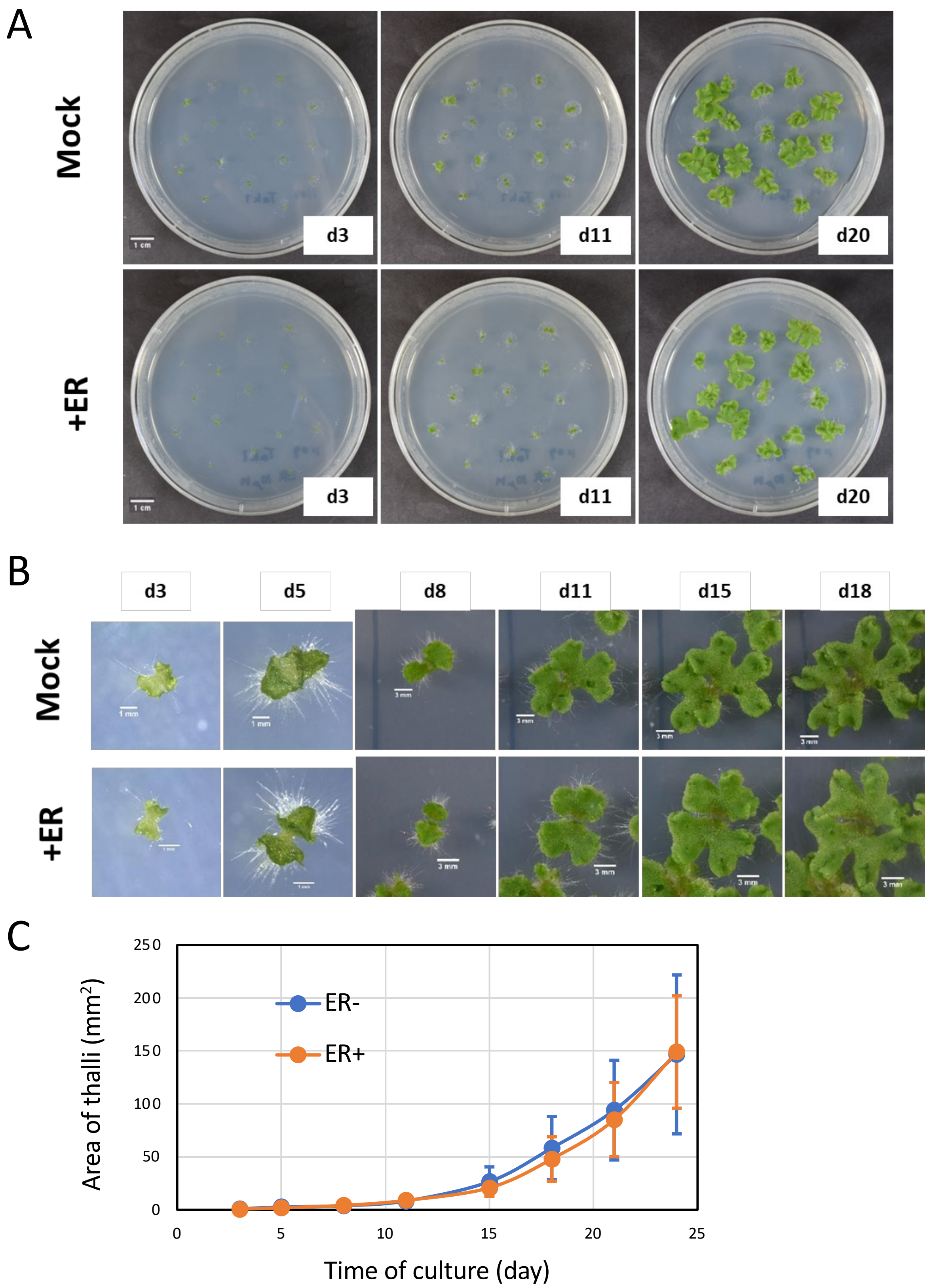

Fig S2. Effect of estradiol on thallus growth of the wild type.

(A) The gemmae of the wild type (Tak-1) were planted in the agar medium with (+ER) or without 10 μM estradiol (Mock) and grown for 3, 11, and 20 days.

(B) Time course of growth of the wild type with (+ER) or without 10 μM estradiol (Mock) .

(C) Quantification of thallus growth of the wild type with (+ER) or without 10 μM estradiol (Mock). The mean projection area of thalli (n = 10 plants) was quantified by ImageJ. Circles and error bars indicate mean values and standard deviations, respectively.

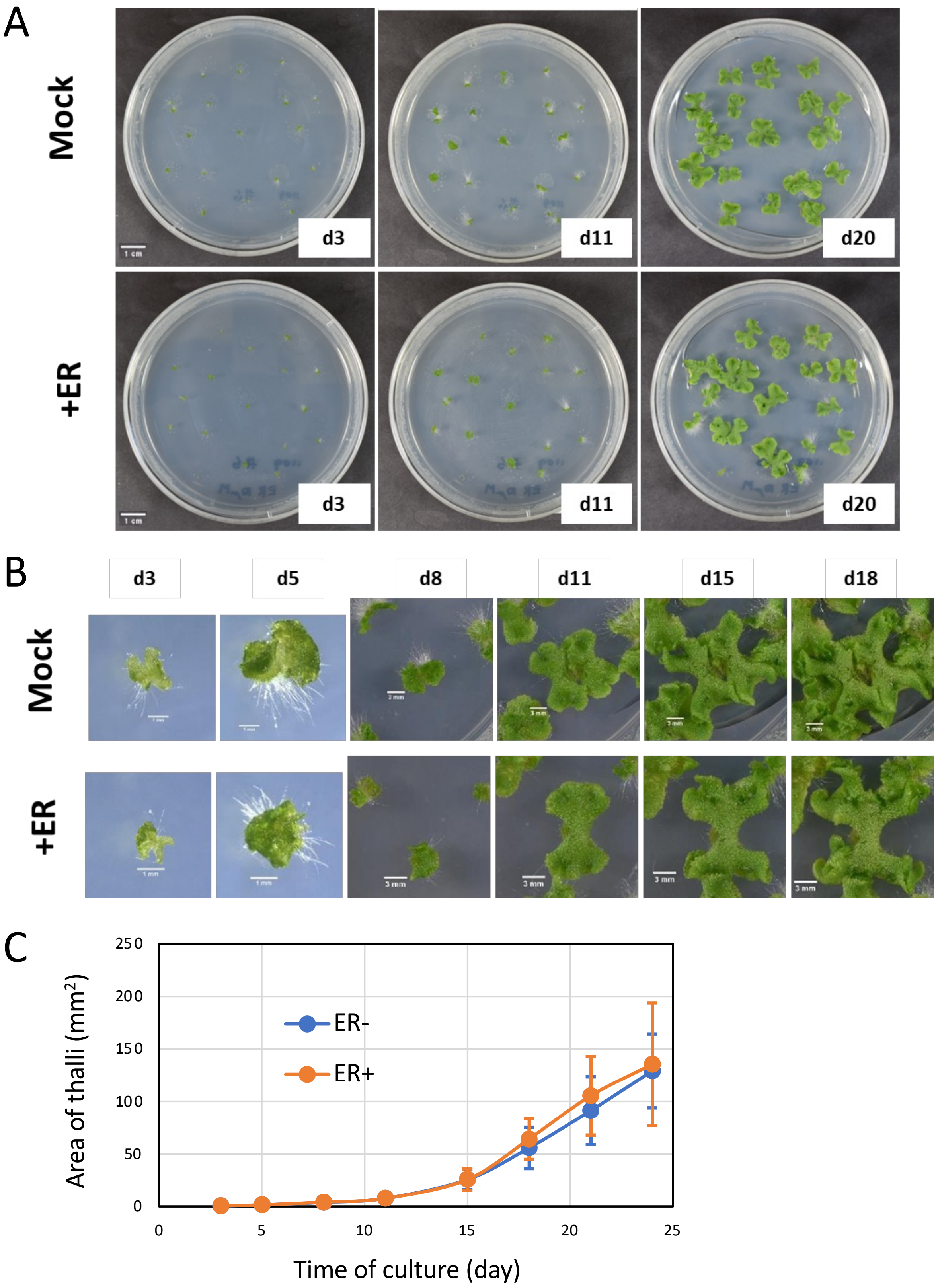

Fig S3. Effect of estradiol on thallus growth of the transgenic line #6 with *XVE::*Mp*NEK1*.

(A) The gemmae of the line (#6) were planted in the agar medium with (+ER) or without 10 µM estradiol (Mock) and grown for 3, 11, and 20 days.

(B) Time course of growth of the line (#6) with (+ER) or without 10 µM estradiol (Mock) .

(C) Quantification of thallus growth of the line (#6) with (+ER) or without 10 µM estradiol (Mock). The mean projection area of thalli ($n = 10$ plants) was quantified by ImageJ. Circles and error bars indicate mean values and standard deviations, respectively.

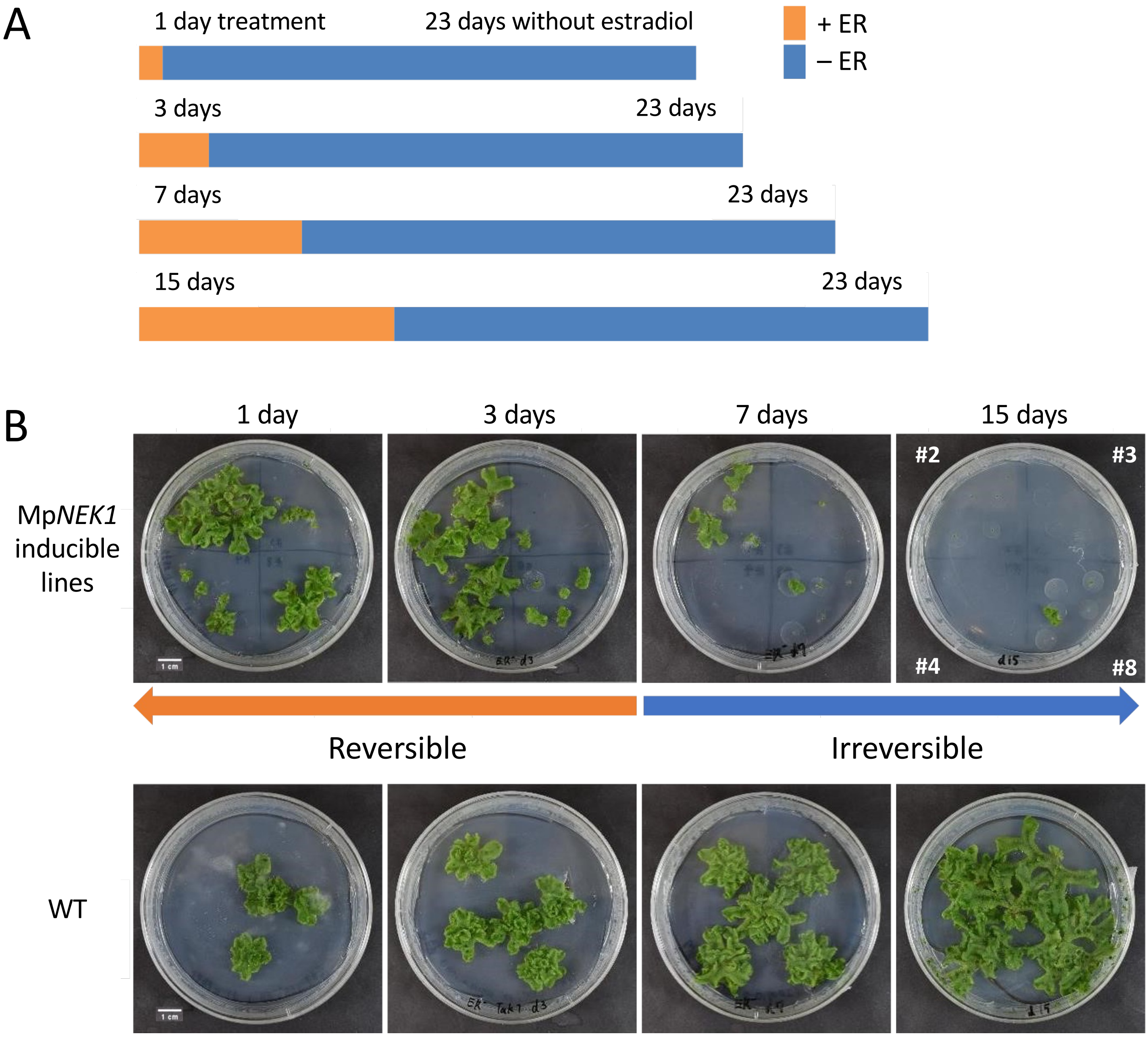

Fig S4. Reversible or irreversible effect of estradiol on thallus growth

(A) Schematic diagram of treatments. Gemmae were planted in the agar medium with 10 µM estradiol, grown in the same medium for 1, 3, 7, or14 days, and then transferred to and grown in the estradiol-free medium for 23 days.

(B) Gemmae of the wild type (WT) and four independent inducible lines (#2, 3, 4, and 8) were grown as shown in (A). The periods of estradiol treatment is shown above the photographs taken at the 23 day after transfer to the estradiol-free medium.

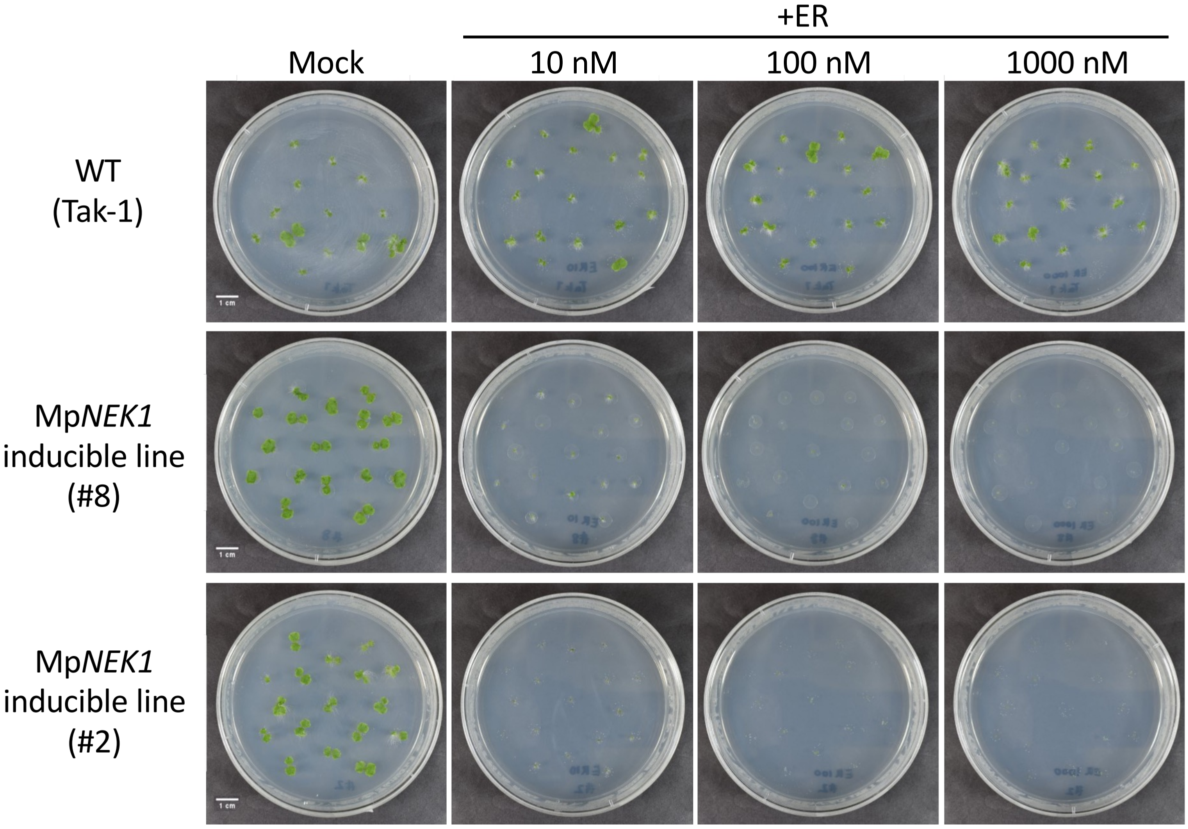

Fig S5. Effect of various concentrations of estradiol on thallus growth.

The gemmae of the wild type and Mp*NEK1* inducible lines were planted in the agar medium supplemented with or without estradiol at the concentration of 10 nM, 100 nM, or 1000 nM and grown for 13 days.

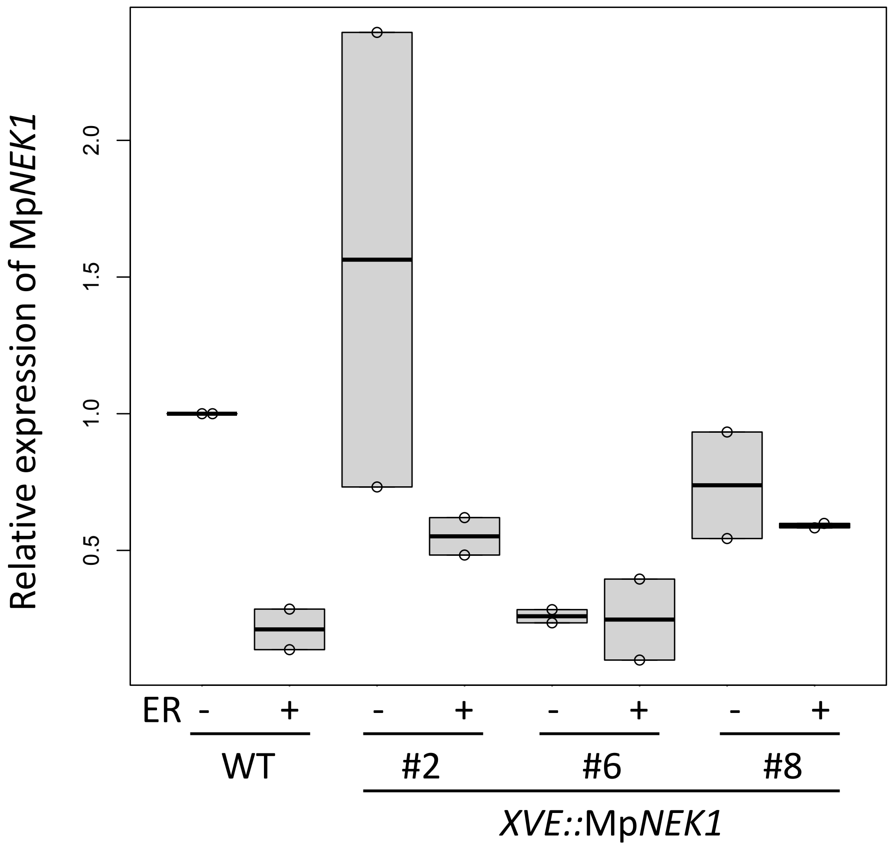

Fig S6. Effect of estradiol on the accumulation of Mp*NEK1* transcripts in the wild type and estradiol-resistant transgenic plants harboring *XVE::*Mp*NEK1*.

The 10-day old plants of the wild type (WT) and estradiol-resistant plants derived from line #2, #6 or #8 were incubated for 1 day in the medium with (+) or without 10 µM estradiol (-). Total RNA was extracted and subjected to RT-qPCR. All transcript levels are relative to that of mock-treated wild type plants. Mp*EF1α* was used as the control genes. Data are shown by box plots (n = 2 biological replicates).

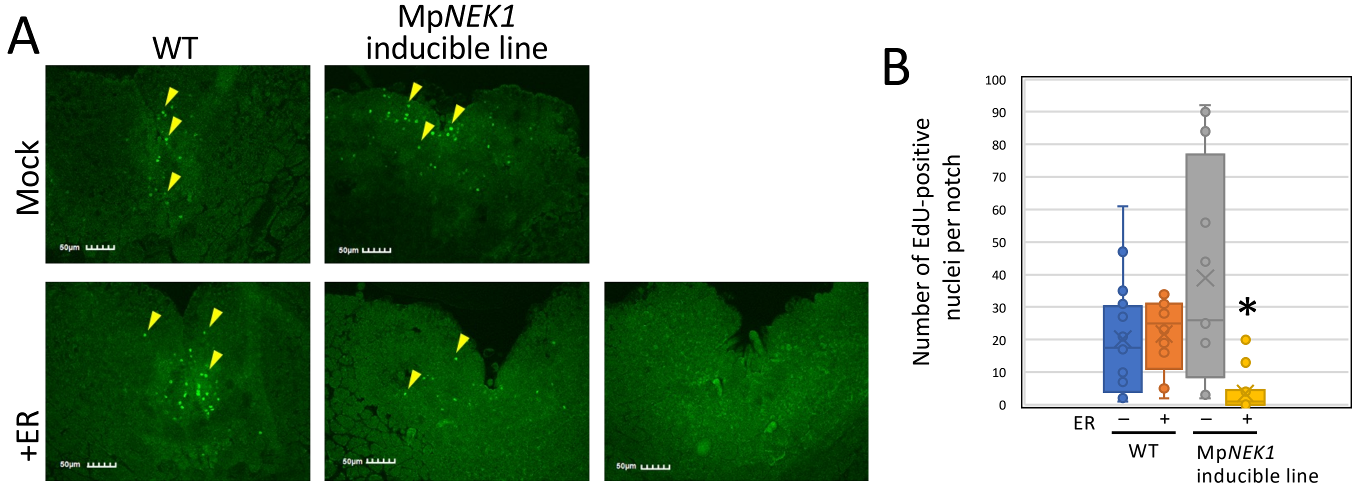

Fig. S7. Effect of estradiol on cell proliferation

(A) EdU-labelled nuclei in the gemmalings treated with 1 μM estradiol for 1 day. The gemmae of the wild type (WT) and Mp*NEK1* inducible line were planted in the medium without estradiol, grown for 3 days, and then transferred to and grown in the medium supplemented with or without 1 μM estradiol for 1 day. The gemmalings were incubated with 10 μM EdU for 1 hour in the same kind of liquid medium. EdU-labelled nuclei were visualized according to the manufacture's instruction as described in methods.

(B) Quantification of EdU-labeled nuclei in the thalli grown as in (A). Data are shown by box plots (n = 12-16 plants). An asterisk indicates significant difference from the control (-ER) (*t*-test, $P < 0.05$).

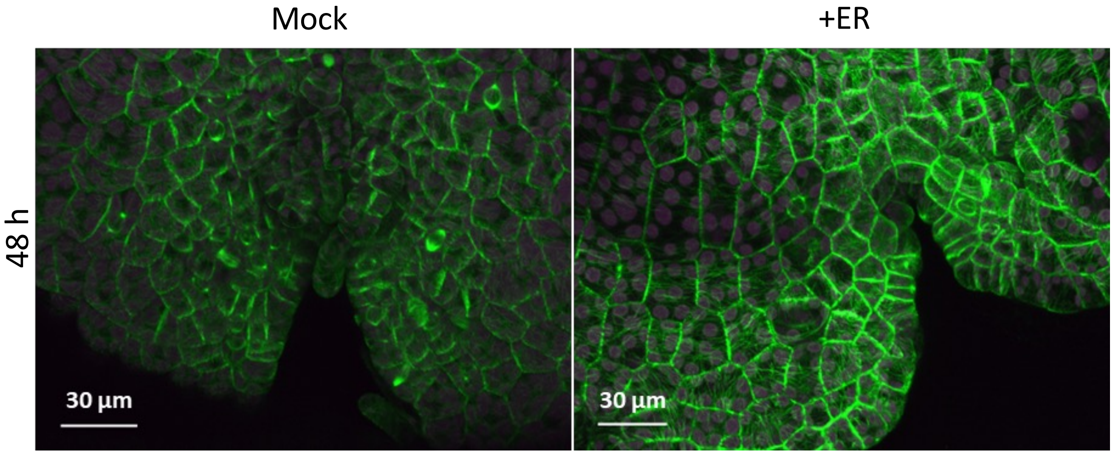

Fig. S8. Effect of MpNEK1 induction on microtubules

The gemmae of MpNEK1 inducible line with a microtubule marker CaMV35S:Citrine-MpTUB2 were grown in the estradiol-free medium for 3 days and then supplemented with the liquid medium with (+ER) or without 10 µM estradiol (Mock). Thalli were observed under a confocal microscope after 48 h of treatment. Green: Microtubule, magenta: plastid autofluorescence.

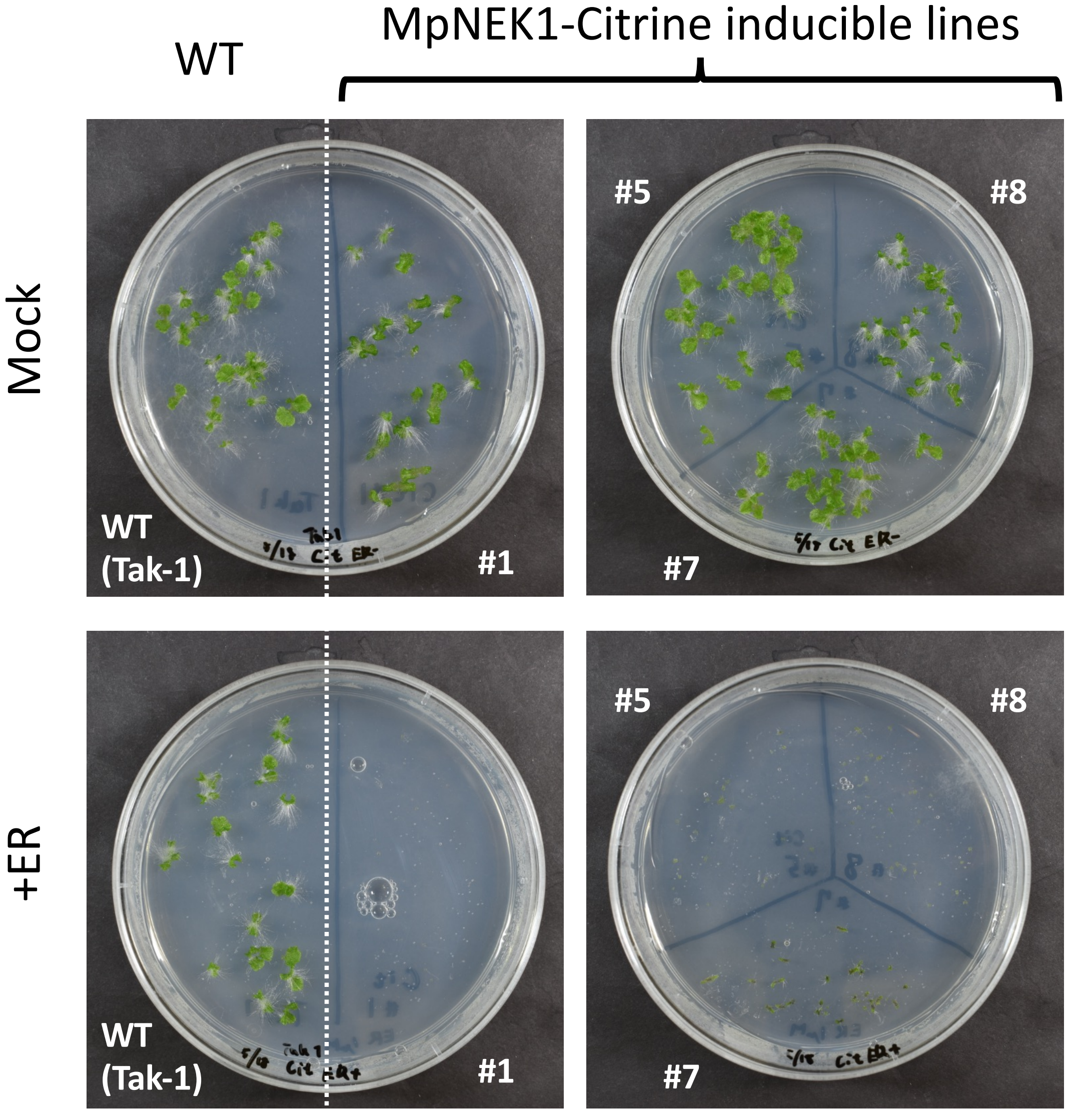

Fig S9. Effect of estradiol on the growth of MpNEK1-Citrine inducible lines

The gammae of the wild type (WT) and the MpNEK1-Citrine inducible lines were planted in the agar medium supplemented with (+ER) or without 1 μM estradiol (Mock) and grown for 15 days.

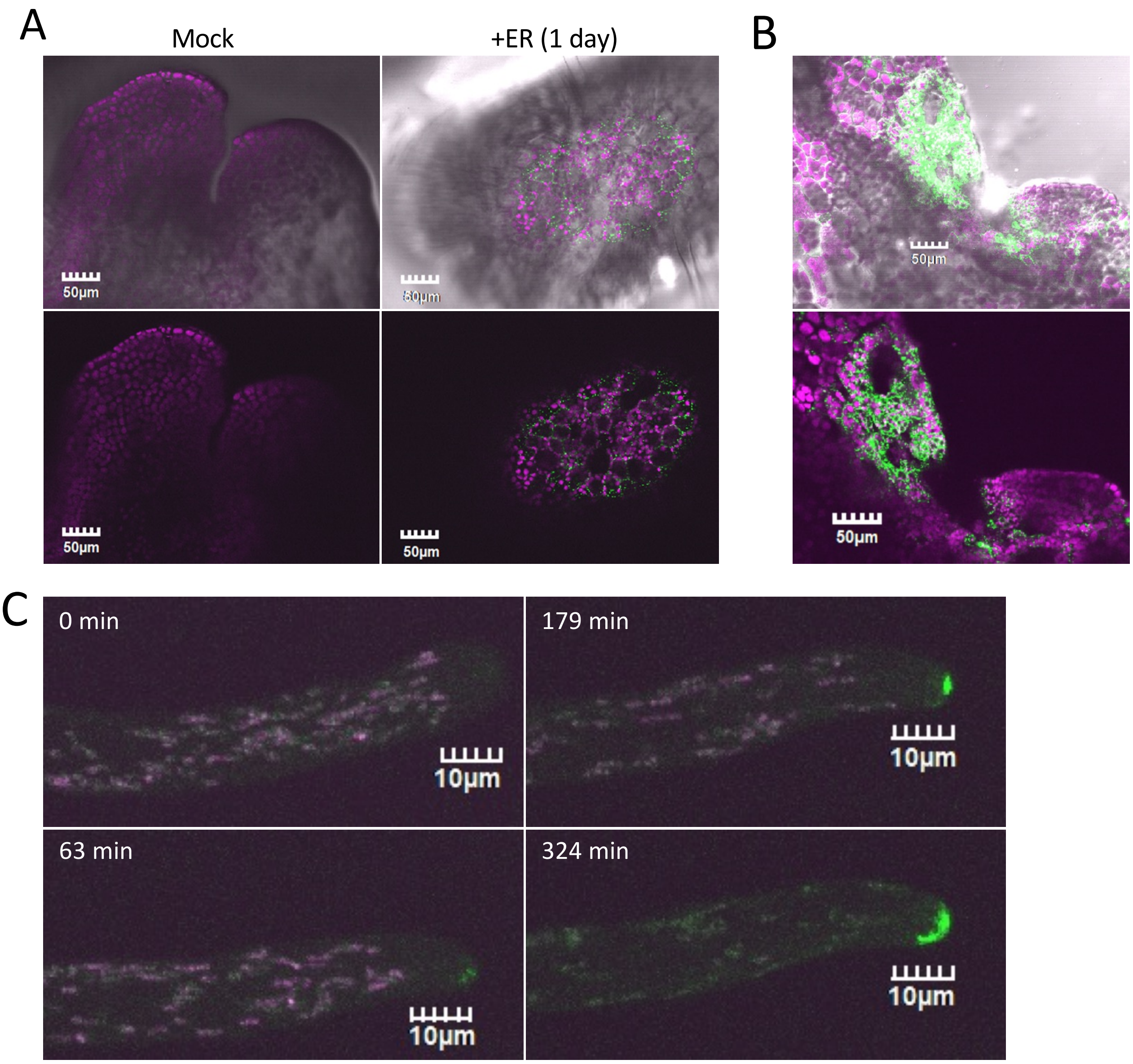

Fig. S10. Localization of MpNEK1-Citrine

(A) Localization of MpNEK1-Citrine in thalli. The gemmae of the Mp*NEK1* inducible line were planted in the medium without estradiol, grown for 2 days, and then supplemented with (+ER) or without 1 µM estradiol (Mock) for 1 day. Upper panels are light field images merged with confocal images shown in the lower panels. Green: MpNEK1-Citrine, magenta: plastid autofluorescence.

(B) Expression of MpNEK1-Citrine in the meristem of thallus without estradiol. Upper panel shows a light field image merged with a confocal image in the lower panel. Green; MpNEK1-Citrine, magenta; plastid autofluorescence.

(C) Localization of MpNEK1-Citrine in a rhizoid. The gemmae of the Mp*NEK1* inducible line were planted in the medium without estradiol, grown for 3 days, and then supplemented with 1 µM estradiol and observed at the time indicated in each panel. The same rhizoid was observed under a confocal microscope over time. Green: MpNEK1-Citrine, magenta: plastid autofluorescence.

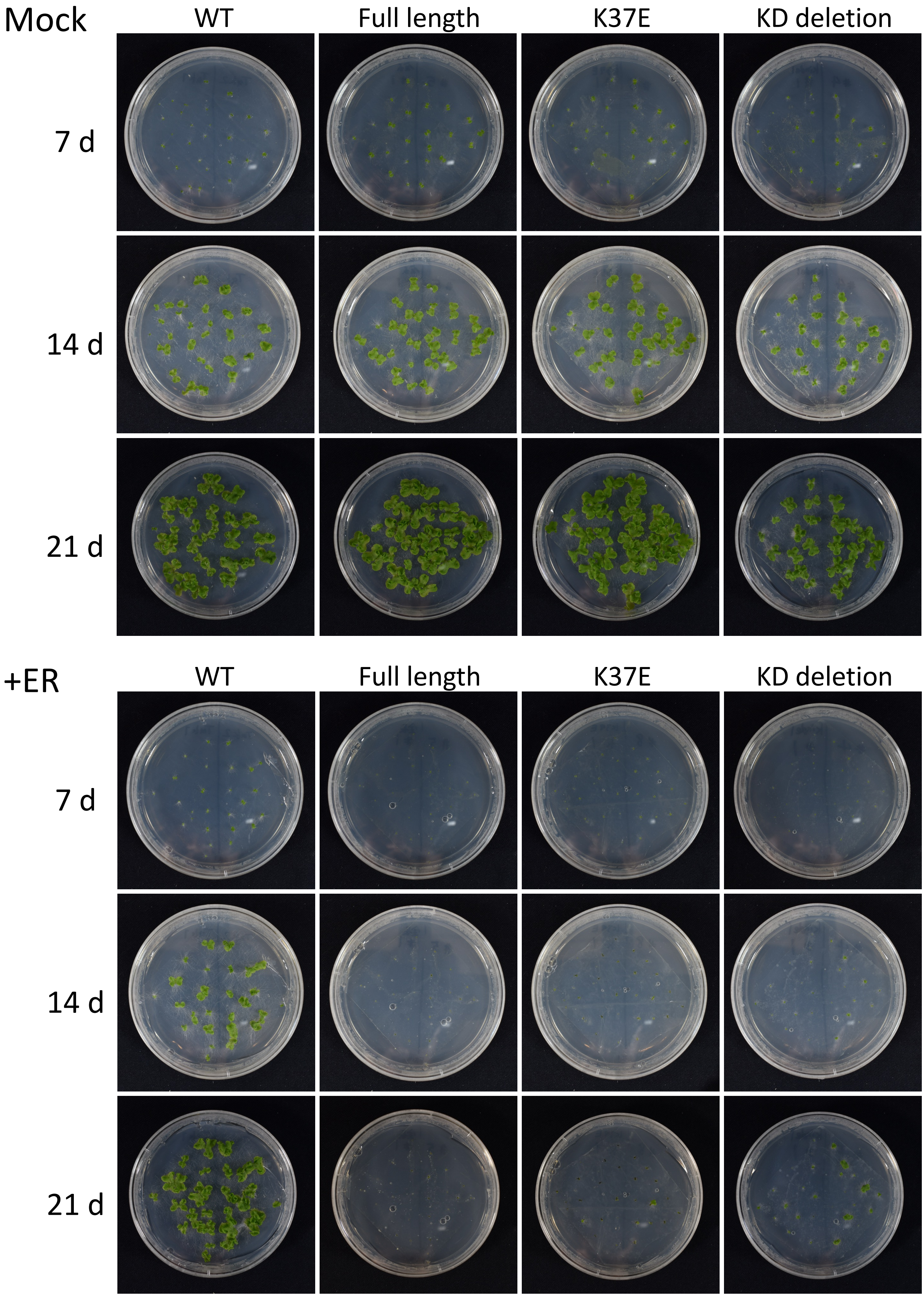

Fig S11. Effect of estradiol on the growth of the wild type and MpNEK1-Citrine inducible lines

The gammae of the wild type (WT) and the MpNEK1-Citrine inducible lines were planted in the agar medium supplemented with (+ER) or without 1 μM estradiol (Mock) and grown for 7, 14, and 21 days.

Video S1. Localization of MpNEK1-Citrine in a gemmaling grown for 4 days in the medium supplemented with 1μM estradiol (z-series of the same plant shown in the right panels of Fig. 7B).

Video S2. Localization and dynamics of MpNEK1-Citine in a rhizoid of MpNEK1-Citrine inducible line treated with estradiol for 24 hours.
MpNEK1-Citrine inducible line was grown without estradiol for 3 days and then supplemented with 1 μM estradiol and grown for 24 hours. Green: MpNEK1-Citrine, magenta: plastid autofluorescence (time lapse of the same rhizoid shown in Fig. 8B).

Video S3. Localization and dynamics of over-accumulated MpNEK1-Citine in a rhizoid of MpNEK1-Citrine inducible line treated with estradiol for 24 hours.
MpNEK1-Citrine inducible line was grown without estradiol for 3 days and then supplemented with 1 μM estradiol and grown for 24 hours. A rhizoid highly accumulating MpNEK1-Citrine was observed. Green: MpNEK1-Citrine, magenta: plastid autofluorescence.

Video S4. Localization and dynamics of MpNEK1-Citine in a rhizoid of MpNEK1-Citrine inducible line treated with estradiol for 48 hours.
MpNEK1-Citrine inducible line was grown without estradiol for 3 days and then supplemented with 1 μM estradiol and grown for 48 hours. Green: MpNEK1-Citrine, magenta: plastid autofluorescence (time lapse of the same rhizoid shown in Fig. 8B).

Video S5. Localization and dynamics of MpNEK1-Citine in a rhizoid of MpNEK1-Citrine inducible line treated with estradiol for 48 hours.
MpNEK1-Citrine inducible line was grown without estradiol for 3 days and then supplemented with 1 μM estradiol and grown for 48 hours. Green: MpNEK1-Citrine, magenta: plastid autofluorescence (time lapse of the same rhizoid shown in Fig. 8D).

Video S6. Localization and dynamics of MpNEK1-Citine in a rhizoid of MpNEK1-Citrine inducible line treated with estradiol for 120 hours.
MpNEK1-Citrine inducible line was grown without estradiol for 3 days and then supplemented with 1 μM estradiol and grown for 120 hours. Green: MpNEK1-Citrine, magenta: plastid autofluorescence (same rhizoid shown in Fig. 8B).

Video S7. Localization of MpNEK1-Citine in a rhizoid of MpNEK1-Citrine inducible line treated with estradiol for 120 hours.
MpNEK1-Citrine inducible line was grown without estradiol for 3 days and then supplemented with 1 μM estradiol and grown for 120 hours. Green: MpNEK1-Citrine, magenta: plastid autofluorescence.
