# Supplementary material for: Involvement of a NIMA-related kinase in cell division of the liverwort Marchantia polymorpha
Source: Plant Cell Physiol. 2025 Feb 17;66(5):815–32. doi: 10.1093/pcp/pcaf021 (PMC12125575; doi:10.1093/pcp/pcaf021)
Supplement: pcaf021_Supp [file pcaf021_supp.zip › suppl_data/pcp-2024-e-00281-File017.pdf]

Table S1. Primers used in this study		
Primer	Sequence	Purpose
MpNEK-F1-RT(50-69)	GCGCCTTTGGATCTGCAATA	RT-qPCR
MpNEK-R1-RT(205-224)	ACCCAGGCTTCCTTGTAGTC	RT-qPCR, colony PCR & sequencing of XVE vectors
MpNEK-F2-RT-rev	TTCCAGCAGAAcGACGACTG	RT-qPCR, colony PCR & sequencing of XVE vectors
MpNEK-R2-RT(2144-63)	GTAGCTCAGGCTGTCCTTGG	RT-qPCR
MpEF1-F1	AAGCCGTCGAAAAGAAGGAG	RT-qPCR
MpEF1-R1	TTCAGGATCGTCCGTTATCC	RT-qPCR
MpACT-F1	AGGCATCTGGTATCCACGAG	RT-qPCR
MpACT-R1	ACATGGTCGTTCCTCCAGAC	RT-qPCR
XVE-after35S-F	ACTCTAGCCTCGAGGCGCGC	Colony PCR & sequencing of XVE vectors
MpNEK-F5	ATGCAGCCGGACTACGACGAG	Colony PCR & sequencing of XVE vectors
MpNEK-F6	TGGACCTGCCCGGCAAGATGG	Colony PCR & sequencing of XVE vectors
Cit-F-InF-AscI	agtaagggtgggcgcgccgacATGGTGAGCAAGGGCGAGGAGCT	In-Fusion cloning of Citrine
Cit-R-InF-AscI	agctgggtcggcgcgTTACTTGTACAGCTCGTCCATGCCG	In-Fusion cloning of Citrine
MpNEK1-F-K37E	gAAAAGATCCGTCTCGCTCGTCAGACG	Innverse PCR-based mutagenesis (K37E substitution)
MpNEK1-R-L36	GAGGACATACTTCTTCTTTTCGAGCT	Innverse PCR-based mutagenesis (K37E substitution)
MpNEK1-F-Ctail+start	atgCAACCGTATATCACTCAGTGCCGGTT	Innverse PCR-based mutagenesis (deletion of kinase domain)
ENTR-D-R(+start)	CATGGTGAAGGGGGCGGCCG	Innverse PCR-based mutagenesis (deletion of kinase domain)
Citrine-R-176	GAAGGTGGTCACGAGGGTGG	Colony PCR & sequencing of XVE vectors
