# Supplementary figures and images for: Involvement of a NIMA-related kinase in cell division of the liverwort Marchantia polymorpha
Source: Plant Cell Physiol. 2025 Feb 17;66(5):815–32. doi: 10.1093/pcp/pcaf021 (PMC12125575; doi:10.1093/pcp/pcaf021)

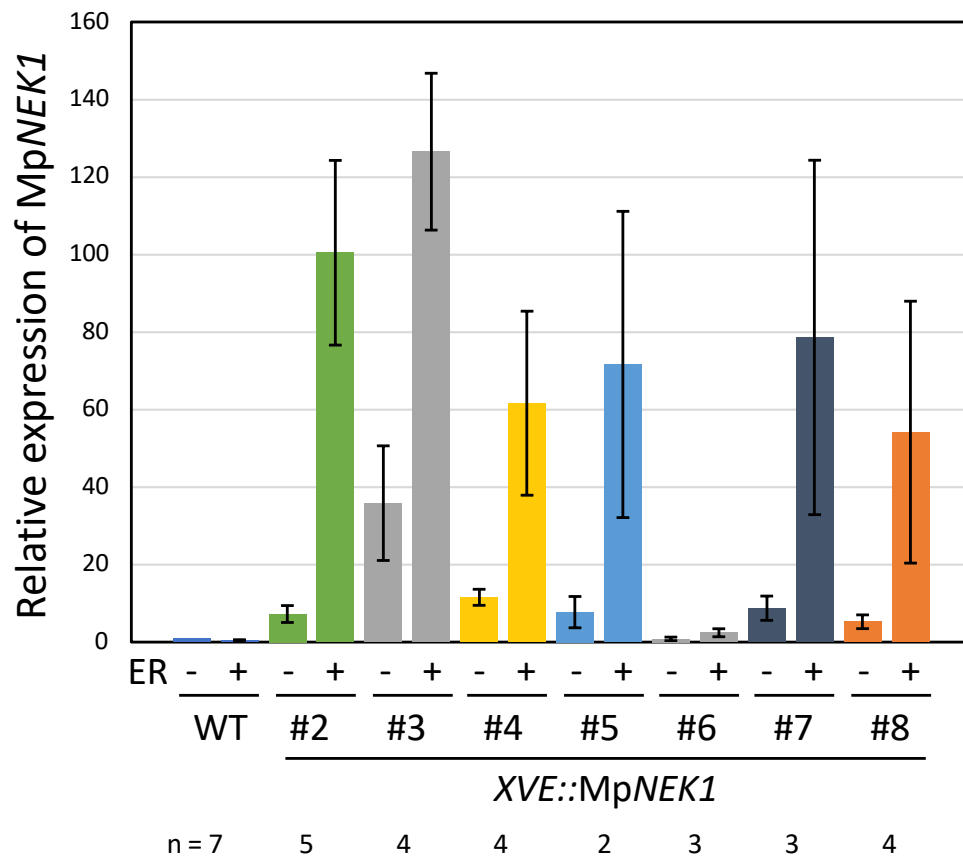

A

Mock

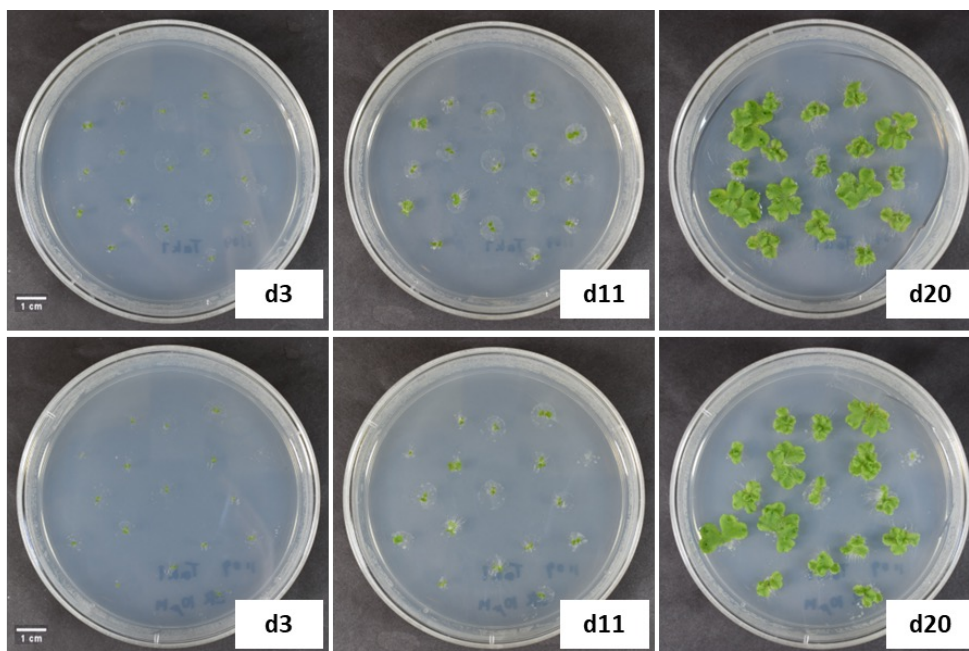

+ER

B

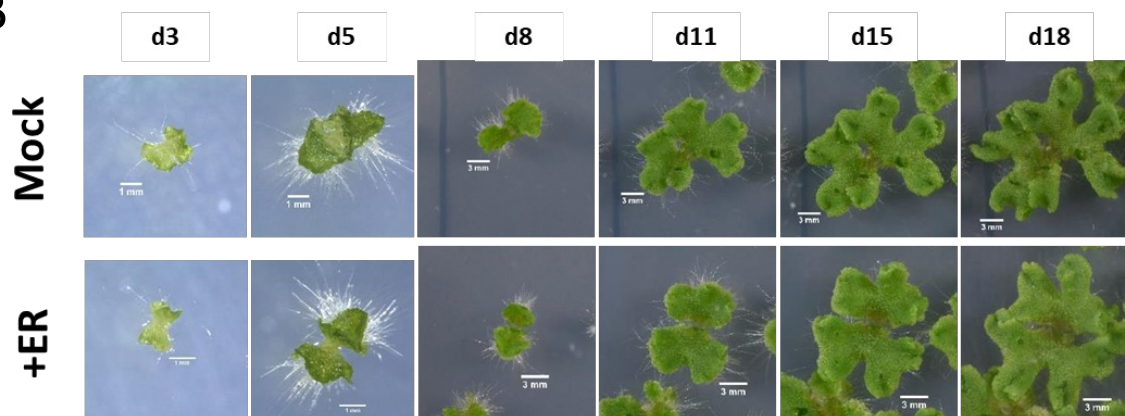

C

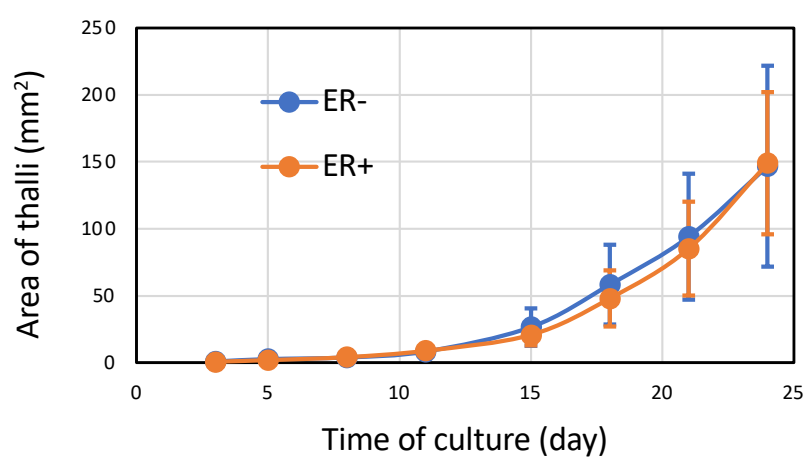

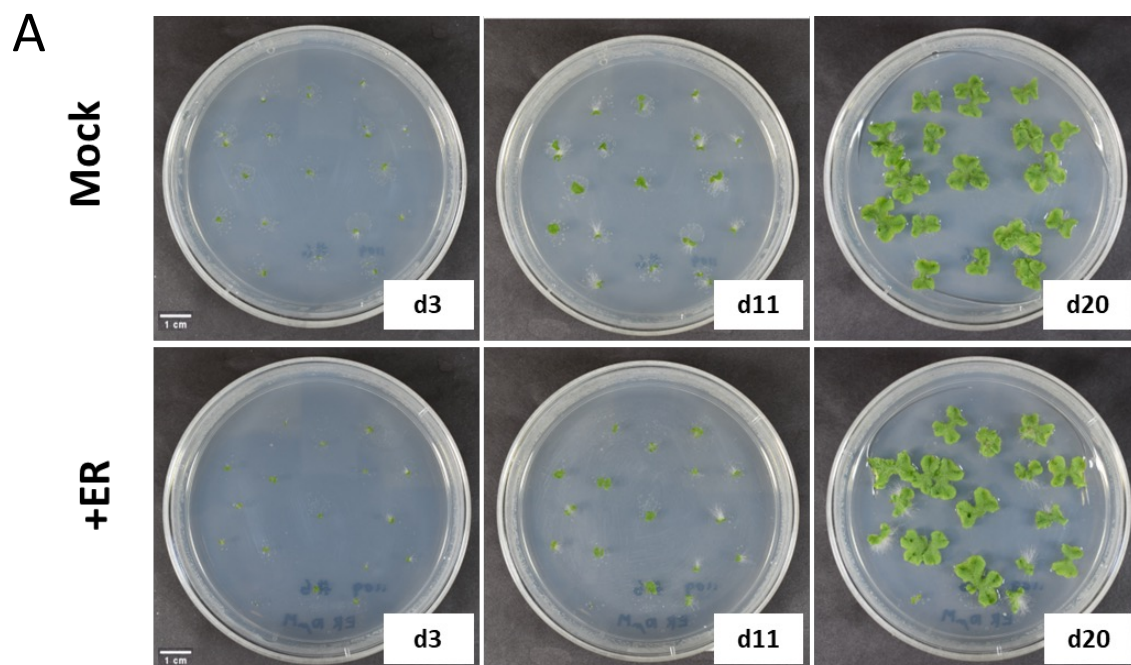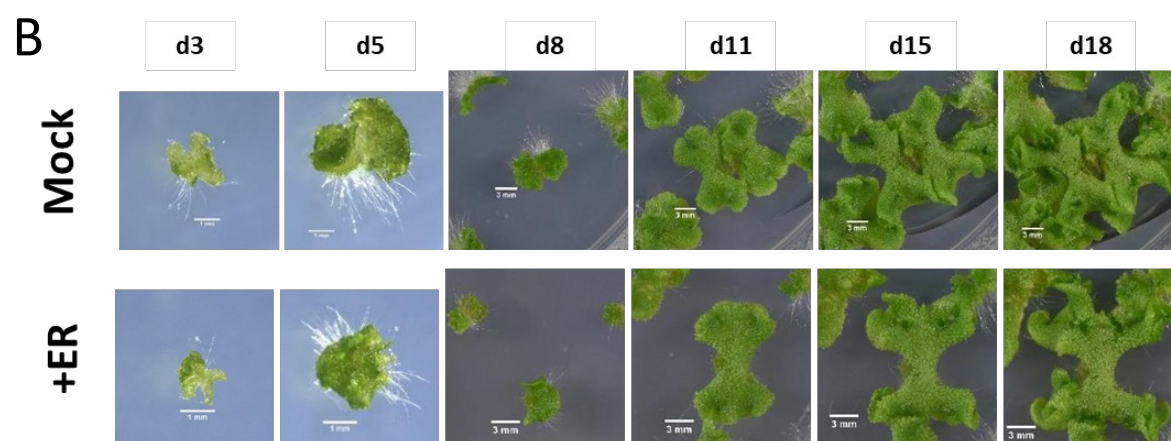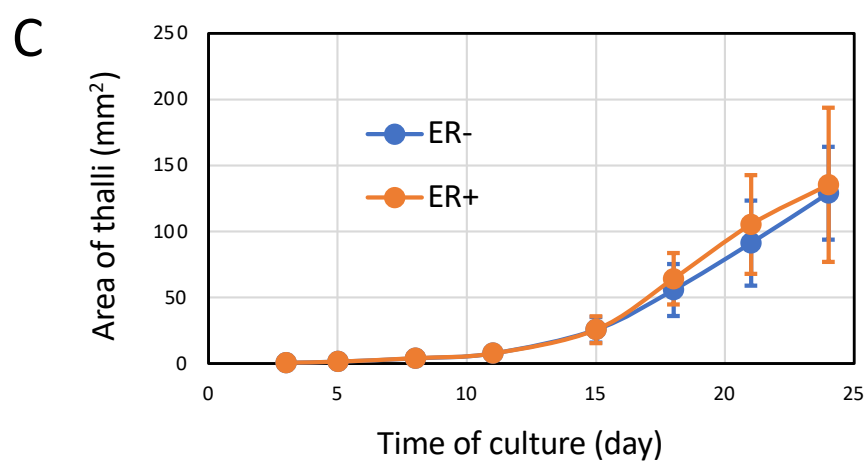

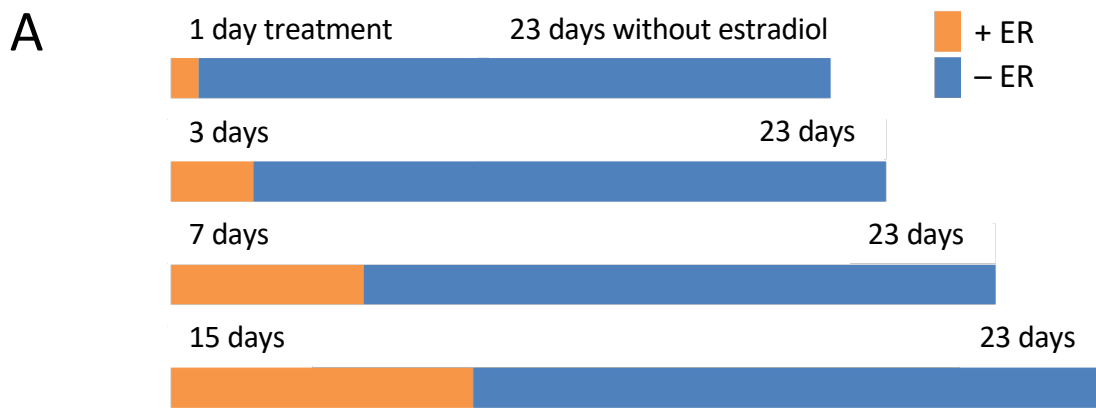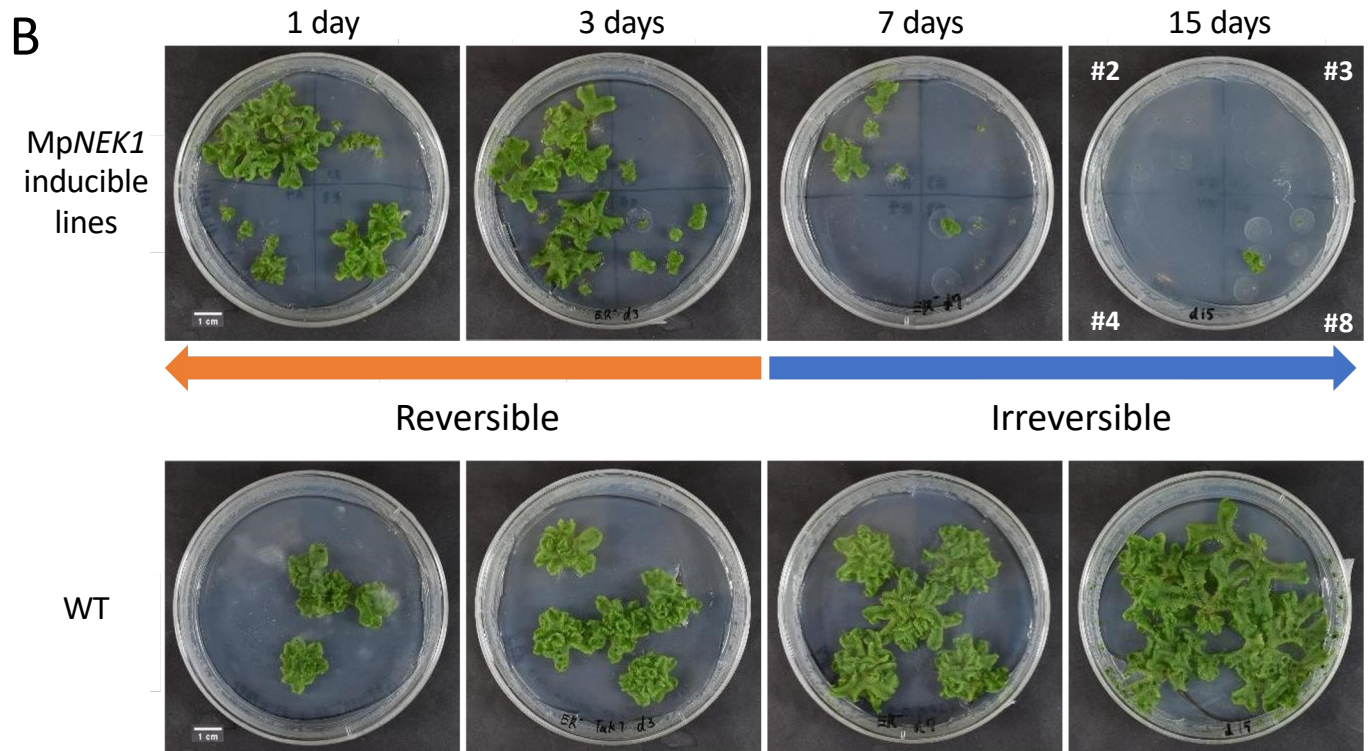

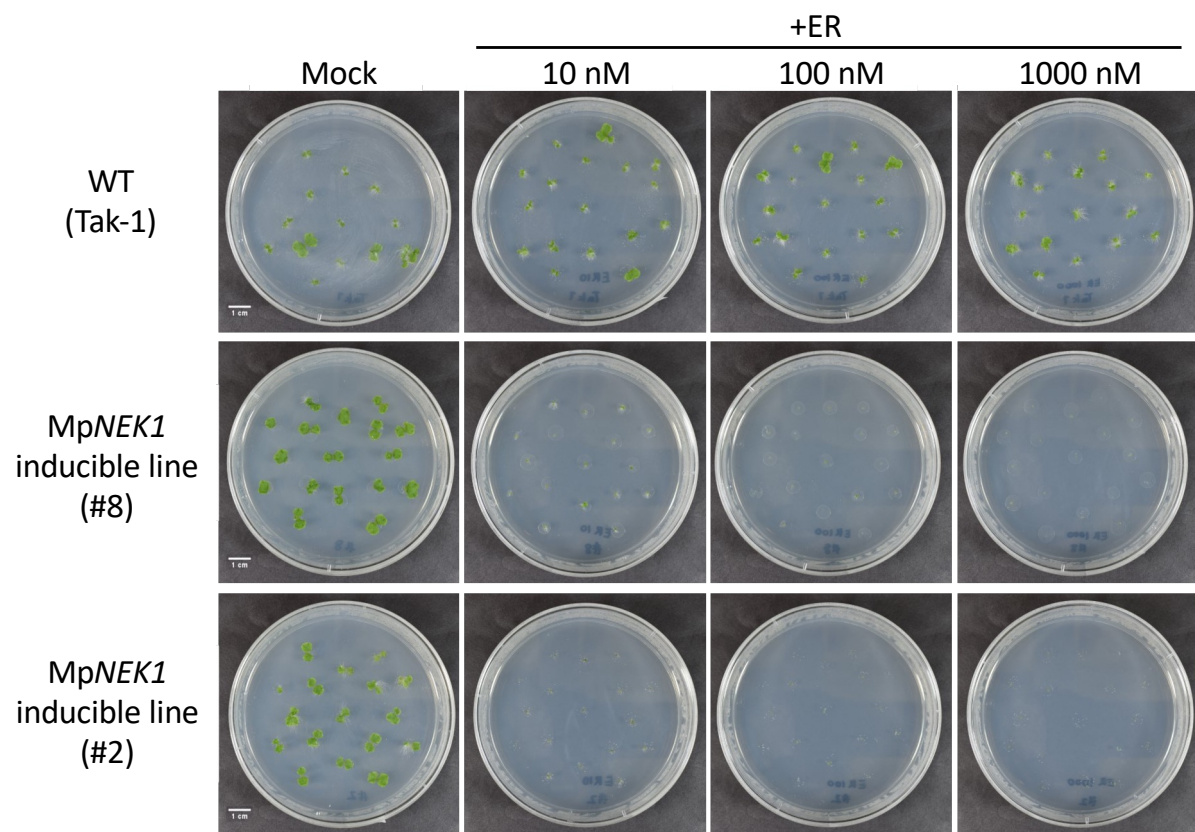

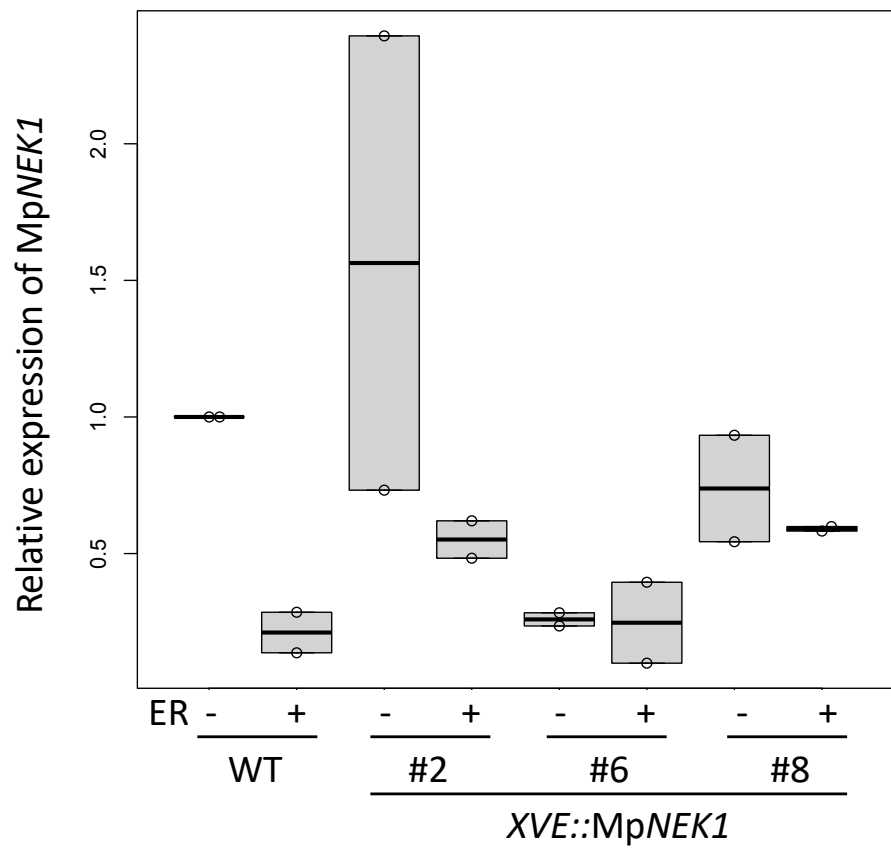

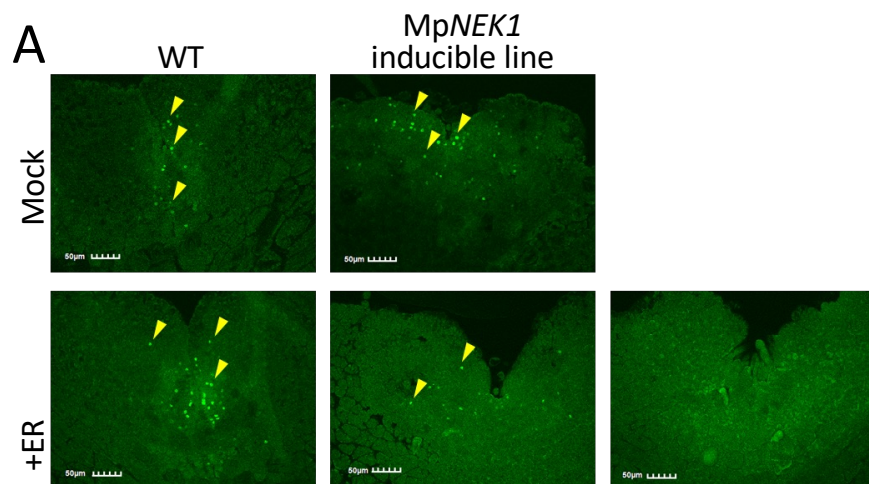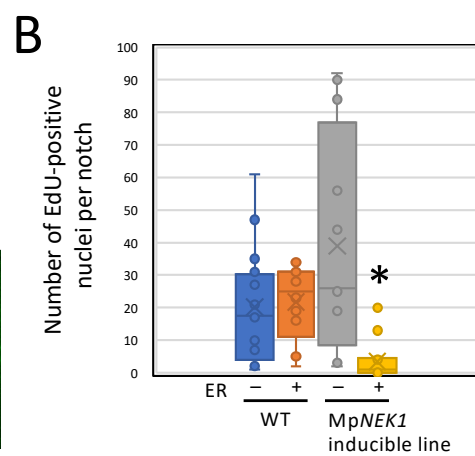

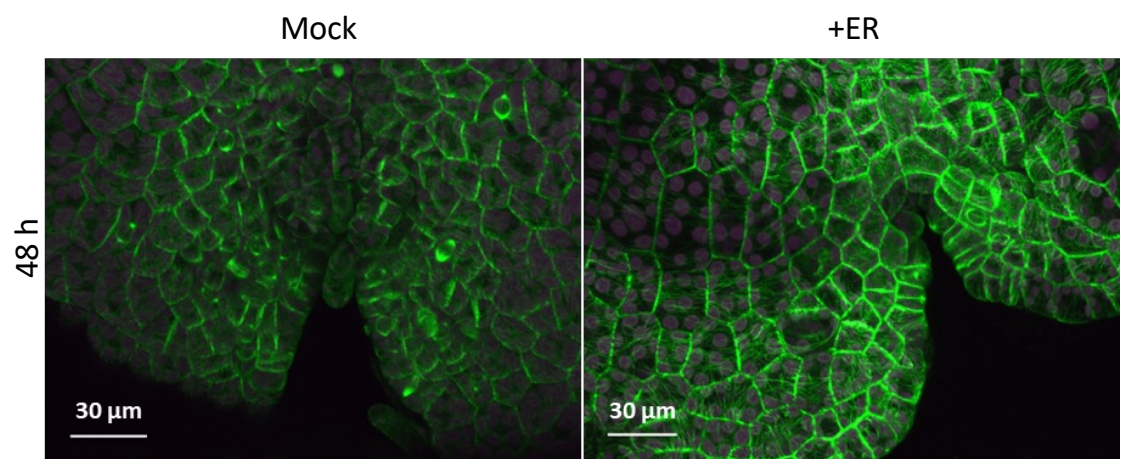

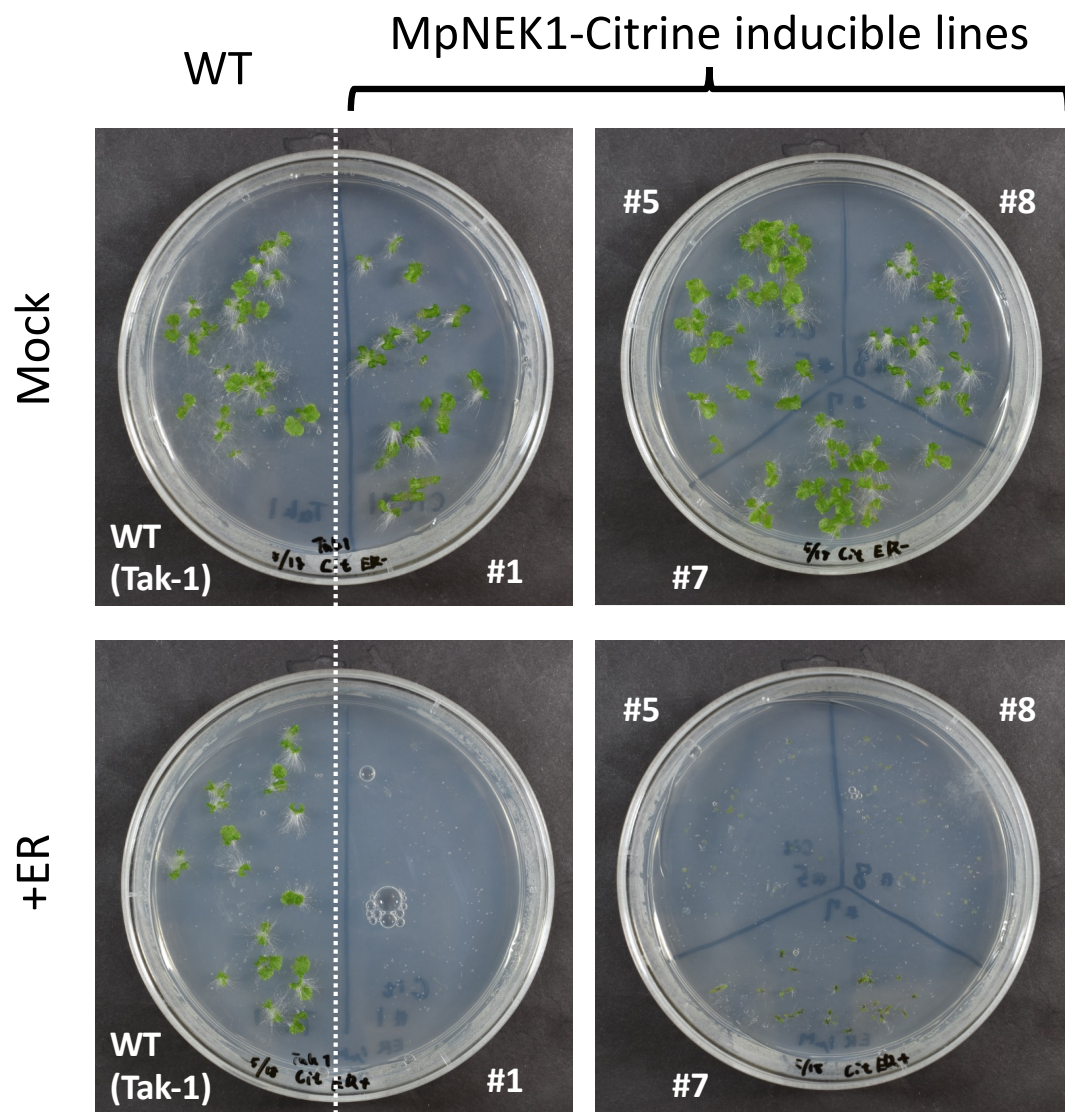

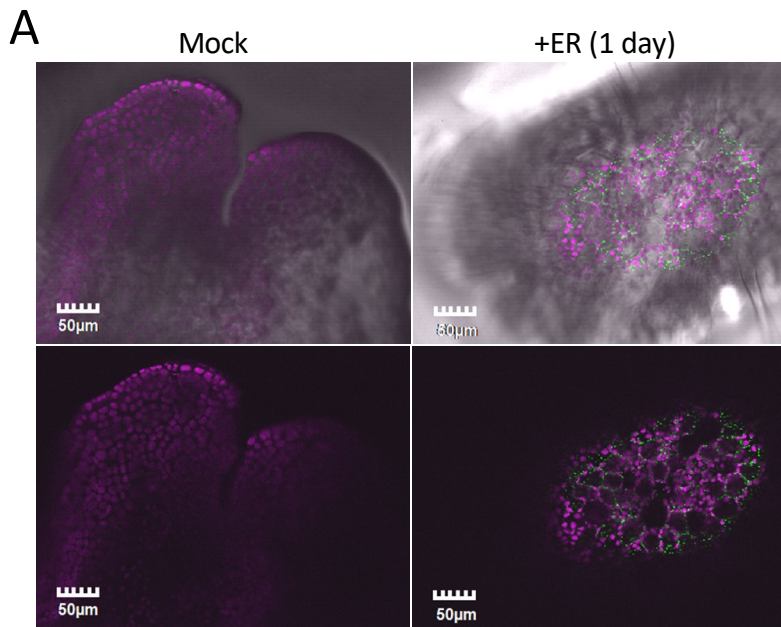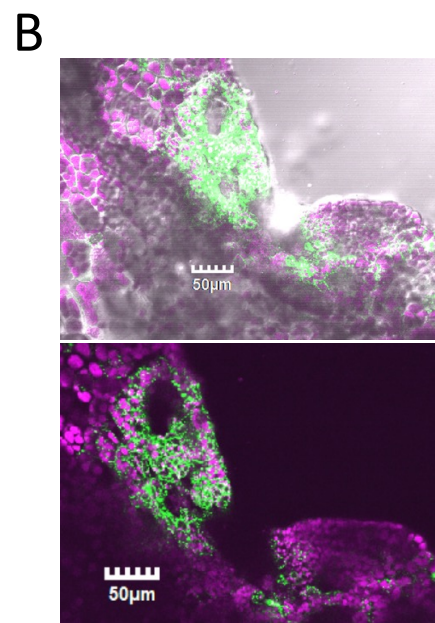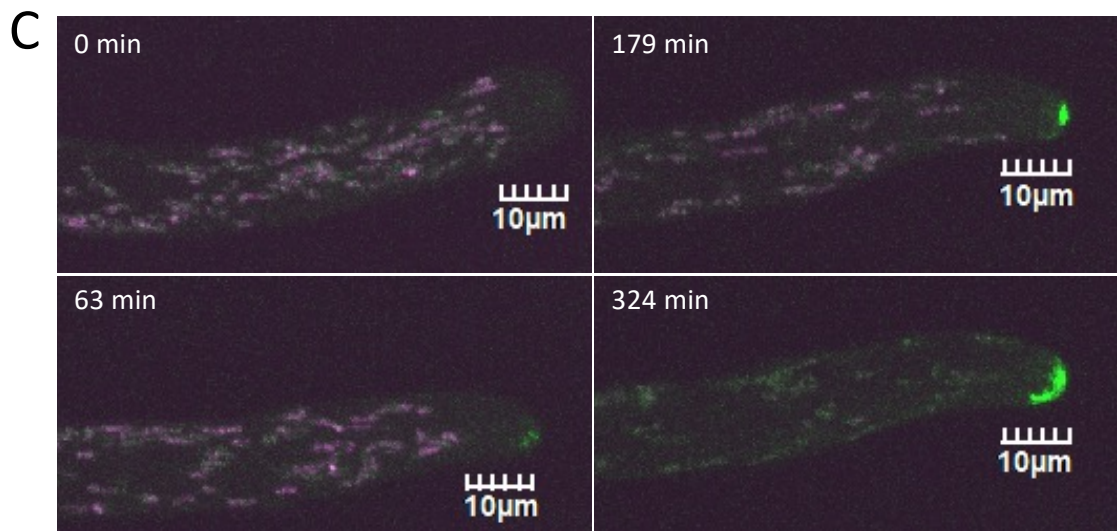

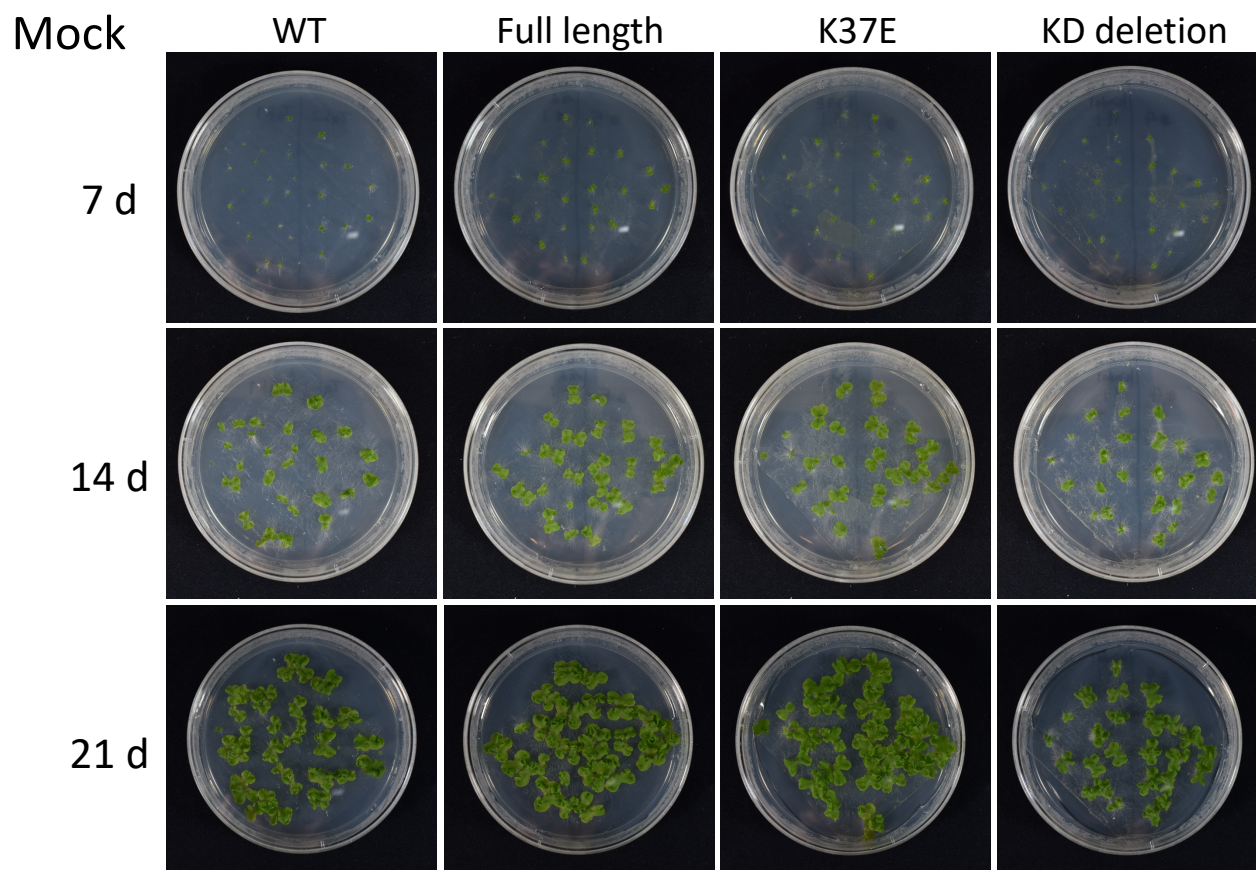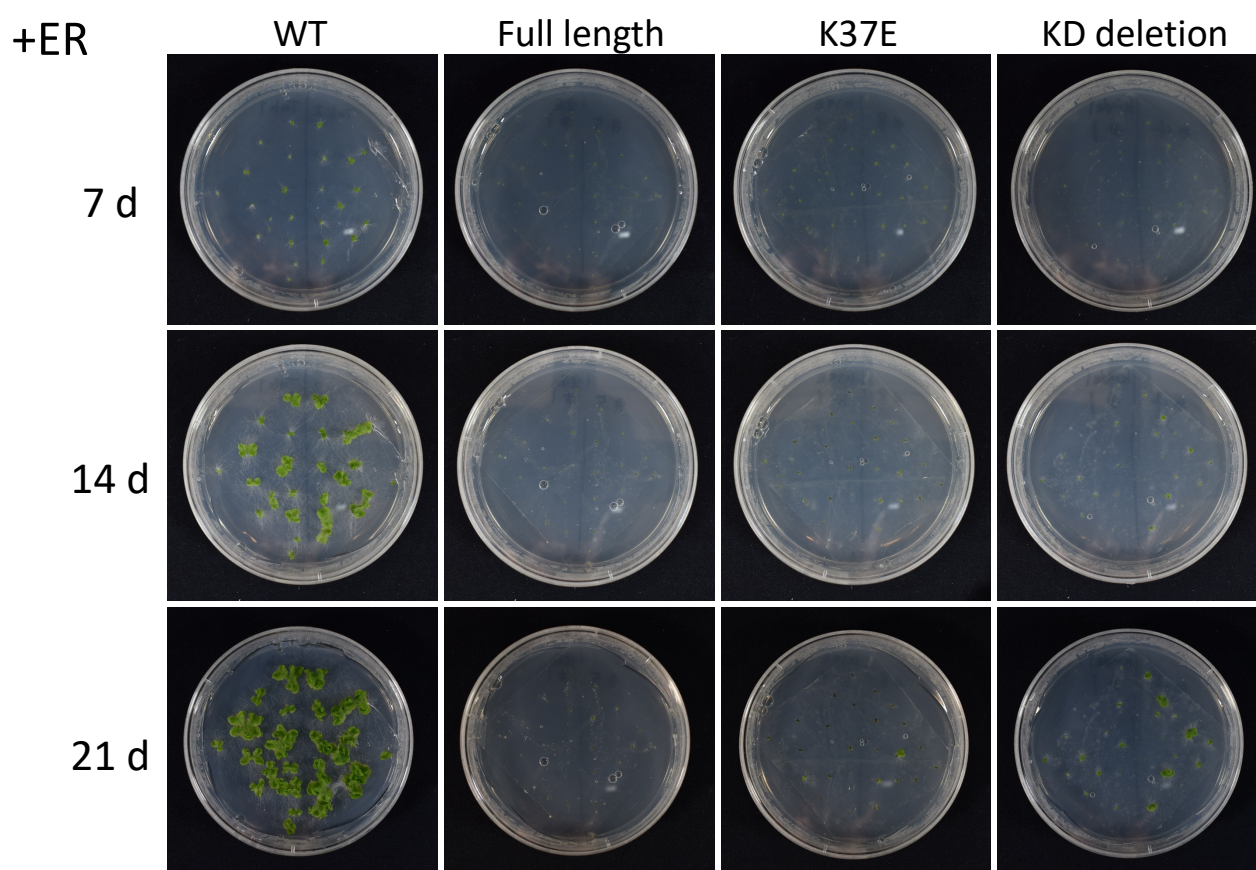

Supplement: pcaf021_Supp [file pcaf021_supp.zip › suppl_data/pcp-2024-e-00281-File015.pdf]
